# AuCFSR: Authentication and Color Face Self-Recovery Using Novel 2D Hyperchaotic System and Deep Learning Models

**DOI:** 10.3390/s23218957

**Published:** 2023-11-03

**Authors:** Achraf Daoui, Mohamed Yamni, Torki Altameem, Musheer Ahmad, Mohamed Hammad, Paweł Pławiak, Ryszard Tadeusiewicz, Ahmed A. Abd El-Latif

**Affiliations:** 1 National School of Applied Sciences, Sidi Mohamed Ben Abdellah University, Fez 30000, Morocco; achraf.daoui@usmba.ac.ma; 2 Dhar El Mahrez Faculty of Science, Sidi Mohamed Ben Abdellah University, Fez 30000, Morocco; mohamed.yamni@usmba.ac.ma; 3 Computer Science Department, Community College, King Saud University, Riyadh 11451, Saudi Arabia; altameem@ksu.edu.sa; 4 Department of Computer Engineering, Jamia Millia Islamia, New Delhi 110025, India; musheer.cse@gmail.com; 5 Department of Information Technology, Faculty of Computers and Information, Menoufia University, Shibin El Kom 32511, Egypt; mohammed.adel@ci.menofia.edu.eg; 6 Department of Computer Science, Faculty of Computer Science and Telecommunications, Cracow University of Technology, Warszawska 24, 31-155 Krakow, Poland; 7 Institute of Theoretical and Applied Informatics, Polish Academy of Sciences, Bałtycka 5, 44-100 Gliwice, Poland; 8 Department of Biocybernetics and Biomedical Engineering, AGH University of Science and Technology, 30-059 Krakow, Poland; rtad@agh.edu.pl; 9 Information Countermeauser Technique Institute, School of Cyberspace Science, Faculty of Computing, Harbin Institute of Technology, Harbin 150001, China; 10 Department of Mathematics and Computer Science, Faculty of Science, Menoufia University, Shibin El Kom 32511, Egypt

**Keywords:** hyperchaotic systems, fragile watermarking, deep learning models, tamper detection, self-recovery, color image authentication

## Abstract

Color face images are often transmitted over public channels, where they are vulnerable to tampering attacks. To address this problem, the present paper introduces a novel scheme called Authentication and Color Face Self-Recovery (AuCFSR) for ensuring the authenticity of color face images and recovering the tampered areas in these images. AuCFSR uses a new two-dimensional hyperchaotic system called two-dimensional modular sine-cosine map (2D MSCM) to embed authentication and recovery data into the least significant bits of color image pixels. This produces high-quality output images with high security level. When tampered color face image is detected, AuCFSR executes two deep learning models: the CodeFormer model to enhance the visual quality of the recovered color face image and the DeOldify model to improve the colorization of this image. Experimental results demonstrate that AuCFSR outperforms recent similar schemes in tamper detection accuracy, security level, and visual quality of the recovered images.

## 1. Introduction

Nowadays, the communication of digital images between people has become both fast and easy thanks to the use of modern communication channels. However, this communication is not always reliable, as the communicated images can be subject to modification/editing attacks during their transmission. These attacks can cause serious issues for individuals and institutions when unauthorized persons manipulate images of sensitive content, such as medical and military images. Indeed, for medical image, even a minimal change in the image’s content can lead to an erroneous judgment from the doctor [1], which in turn can lead to harmful consequences on the patient’s health. As well, face images can be subject to manipulation and tampering during their transmission through different communication channels. There are many tools and software that can be easily used by users, including non-professional ones, to manipulate facial images. The tampered face images can then be misused for diffusing fake news and misinformation [2]. Fragile watermarking is an excellent method that can be used to protect the authentication of digital images communicated via the Internet. It is designed to be easily destroyed or corrupted by any unauthorized modifications to the image, making it a good way to detect tampering. Recent fragile watermarking schemes [3,4,5,6] not only detect the tampered areas with high accuracy, but also recover damaged pixels of the tampered areas.

To accurately locate tampered image by fragile watermarking schemes, a watermark is embedded into the host image. The watermark is a unique identifier that can be used to identify the original image and detect tampering. The recovery data is also embedded into the host image. The recovery data can be used to restore the original image if it has been tampered with. Both the watermark and recovery data are usually embedded into the least significant bits (LSBs) of the host image pixels. LSB-based watermarking is considered efficient in terms of computational complexity and provides good imperceptibility, meaning that the watermarked image is visually indistinguishable from the original image [3,7].

Fragile watermarking methods can be divided into two types: block-based and pixel-based [8]. For pixel-based watermarking schemes, the watermark and the recovery data are inserted into individual pixels of the host image [9]. While, for block-based watermarking [3,4,8,10], the host image is subdivided into small-sized blocks (i.e., 2×2 or 3×3). Then, the watermark bits and the recovery data are embedded into these blocks. In the block-based watermarking, each block consists of a number of pixels. If only one pixel from this block is tampered with while the others are not, all the block pixels are considered as tampered data [9]. This problem leads to a lower accuracy in the tamper detection rate. To overcome this problem, pixel-based watermarking schemes can be involved as these schemes detect the tampered areas from the image pixels, which guarantees a high accuracy rate of such schemes. Another problem related to the image authentication based on fragile watermarking is the tampering coincidence problem [11]. This problem appears when the recovery data is corrupted by attackers, which makes it impossible to retrieve the content of the tampered areas [8]. However, this problem can be solved by using image inpainting techniques, which aim to replace the tampered image areas with realistic content [12]. Inpainting of images can be performed using various approaches as described in [12,13,14]. Among the existing methods, the deep learning-based ones show very promising results [13]. Indeed these methods show high performances in recovering corrupted data of small holes in low-resolution images, as well as regular structure shapes [14]. However, inpainting of complex shaped textures, large-sized irregular holes and high-resolution images are the main hotspots that deserve special attention in image inpainting techniques research [14,15]. To overcome this issue, the current paper introduces a new approach for image inpainting in blind fragile image watermarking. This approach is a part of the proposed AuCFSR scheme.

AuCFSR scheme initially embeds a binary watermark in the LSB of each RGB image pixel. This watermark is produced based on the proposed 2D-MSCM, which shows an excellent hyperchaotic behavior and superiority over other recent hyperchaotic maps. Next, 2D-MSCM is used to securely embed the recovery data into the two LSBs of each image pixel. This recovery data is extracted from the MSBs of the cover image. Finally, the AuSFR output image can be safely transmitted over a public communication channel.

To detect tampering areas and recover their contents, AuSFR performs the following stages. The first consists in locating the tampered regions in the protected image. For this task, the watermark bits are extracted from this image and compared to the original binary chaotic watermark that is constructed by 2D-MSCM. After detecting the tampered regions, they are removed (replaced with zero values) from the tampered image, which avoids the tampering coincidence problem. Then, 2D-MSCM is used to generate the recovery image from the non-tampered image regions in order to fill in the cropped areas.

Considering that each RGB pixel in the recovery image is represented by 5 MSBs in our scheme, while 19 bits of this pixel are missing. The reason for this limitation is the proposed approach’s capacity to insert only 5/24 bits per pixel (bpp) of the recovery data. To enhance the quality of the self-recovered image, the proposed AuSFR implements a post-processing phase. In this phase, two deep learning models are utilized. The first one, called CodeFormer [16], is a pre-trained face prediction network. The second model, called DeOldify [17], is an open-source model used for automatic image colorization. Both models contribute to improving the visual quality and color of the recovered image. To demonstrate the validity of our scheme, it is applied to color face images, which contain critical details (nose, eyes, etc.). Nevertheless, the framework presented in this paper remains applicable to generic color images. The major contributions of this work can be highlighted as follows:

- Introducing a new 2D hyperchaotic system called 2D-MSCM.
- Analyzing the chaotic behavior of 2D-MSCM and demonstrating its superiority over similar maps.
- Introducing of a new approach for color face image authentication and self-recovery (AuCFSR) based on 2D-MSCM chaotic system and deep learning models.
- AuCFSR incorporates the watermark and recovery data into 2-LSB of the cover image, which ensures high visual quality of the AuCFSR output image.
- The suggested 2D-MSCM ensures the high security level of the proposed AuCFSR, as its security keys are very sensitive to any variation by +/−10^−15^.
- AuCFSR is a pixel-based system, which guarantees a high precision in tampering detection process.
- The use of deep learning models in the post-processing leads to improving the visual quality of the recovered color face image.
- To the best of our knowledge, AuCFSR is the first authentication and self-recovery scheme designed for color face images.

The rest of this work is organized as follows. The second section outlines the related work. The third section presents the proposed 2D-MSCM and its analysis. The fourth section presents a detailed description of the proposed AuSRCF scheme. The fifth section presents simulations and comparative analyses that demonstrate the effectiveness of our approach.

## 2. Related Work

This section presents a brief literature review of image tampering detection with self-recovery schemes. The related works are summarized in Table 1, which includes the main characteristics of the related work schemes. These characteristics include the development domain of the application scheme (spatial, transformed, or hybrid), the data embedding method (block-based/pixel-based), the data embedding locations in the input image pixels with a depth of 8 bits, the input scheme image category (gray scale, color, etc.). Other important features of the reported work are also presented, including the handling of the image coincidence problem, the analysis of the security level, as well as the integration of deep learning techniques.

From Table 1, we can conclude that the majority of the presented schemes are implemented in the spatial domain [3,4,8,18,20,22,26,27] in preference to the transform or the hybrid domains [19,21,23,25,27]. Indeed, the spatial domain is preferable for designing schemes of reduced complexity that can be easily implemented and quickly executed. On the other hand, the transform domain is more appropriate for designing schemes of good robustness to various attacks (filtering, noise, cropping, compression, etc.). These properties are desirable for image copyright protection based on robust watermarking and zero-watermarking schemes. In image tampering detection schemes, any small change in the image pixels due to manipulations (filtering, compression, etc.) should be considered as a tampered image. For this reason, the spatial domain is more suitable because the image pixels are directly manipulated in the spatial domain via fragile watermarking schemes.

The literature review also shows that existing schemes divide the input image into non-overlapping small-sized blocks and then conduct the image watermarking, tamper detection and image recovery processes based on these blocks. The decomposition of the input image into a set of blocks is generally carried out to reduce the complexity of the algorithms and to carry out the image transformation. However, the subdivision of the input image into blocks leads to visual blocking artifacts in the reconstruction of these blocs in the transform domain [28]. Furthermore, if only one pixel in the block is tempered by unauthorized persons, the other block pixels are considered as tampered one, resulting in a significant false positive detection problem [3]. These issues should be considered when designing new schemes authentication and self- recovery schemes.

Table 1 also shows that the authentication and recovery data are often inserted into the first 2 (or 3) LSBs of the image pixels or into the transformation coefficients. This is evident because changing the pixels/coefficients LSBs results in a minor degradation in the host image imperceptibly. Indeed, the average of the peak signal-to-noise ratio (PSNR) criterion of the [3,4,20,21,22,23,25,26,27] schemes results in watermarked images with a PSNR greater than 44 dB by changing the first 2 LSBs. However, by changing 3 LSBs (or more) results in a decrease in the PSNR values of the output image, as in the case of the schemes [18,19], which lead to achieve watermarked images with PSNR values less than 40 dB via the modification of 3 LSBs. Also, the tampering coincidence problem is often taken into account when designing recent schemes [4,25,26] as the recovered image should not contain visual information from tampered areas. Furthermore, this problem is aggravated when the tampered area is of high proportion [3,4]. This problem should therefore be a focus in the conception of new tampering detection and recovery algorithms. The literature review also shows that an important aspect, namely the security standard, is even neglected or insufficiently addressed in existing schemes, which can make such schemes vulnerable to cyber-attacks. Thus, when designing new schemes, the security aspect should be one of the main concerns in image tampering detection and self-recovery.

The great development in deep learning techniques and the outstanding achievements of these techniques in various fields lead researchers to consider the ways of how to efficiently integrate these techniques in image tampering detection and self-recovery. Recently, Rezaei et al. [27] have successfully integrated a deep learning model into image tampering detection and self-recovery application. In the authors work, a CNN model, namely VGG-16 network is used for generating the watermark bits for image authentication. Moreover, a CNN-based End-to-End compression framework [29] is used to compress the recovery data with possible improvements in the recovered image quality. However, the authors scheme suffers from the problem of the relatively inaccurate detection of the tampered areas, because this scheme is “block-based” with the size of each block is 16×16, which can lead to high false positive rate problem. In addition, the security scheme analysis is not provided in this paper.

To overcome the limitations of existing image tamper detection and self-recovery schemes, the present work proposes a new scheme for color face image tampering detection and self-recovery, which exhibits the following advantages:

- The proposed scheme is pixel-based, which can provide high accuracy in detecting the tampered areas. Therefore, an improvement in the tampering detection accuracy is expected by using the proposed scheme.
- The proposed method integrates the watermark and recovery data into 2 LSBs of each pixel. Therefore, our method ensures low degradation of the host image.
- Our scheme uses the pseudorandom property of the proposed 2D-MSCM to construct the watermark data and to embed the latter with the recovery data into the input image. In addition, the chaotic property of 2D-MSCM is exploited to overcome the problem of tampering coincidence. To the best of our knowledge, this is the first exploitation of chaotic systems in overcoming this problem.
- The proposed scheme involves a post-processing stage that relies on pre-trained deep learning models for improving the recovered image quality. Therefore, an improvement in the quality of the recovered image is expected via our scheme over the latest state-of-the-art schemes.
- The performance of our system is illustrated by its application to the tampering detection and self-recovery of color face images. To the best of our knowledge, this is the first attempt to address such specific problem in the image authentication and self-recovery application.
- The robustness of the provided scheme against brute force attacks and the sensitivity of the security keys are investigated to prove its high level of security.

## 3. Novel 2-D Discrete Hyperchaotic Map and Its Analysis

This section presents a novel 2D discrete hyperchaotic map called modular sine-cosine map (2D-MSCM), which exhibits great dynamical characteristics and features. The states of the proposed map govern the following mathematical model ($f_{2D-\mathrm{MSCM}}(\varepsilon ,\beta ,c)$) described in Equation (1).

(1) f2D-MSCM(ε,β,c)=x(n+1)=ε.sinπ(x(n)+y(n).coscx(n)+β.x(n)mod1y(n+1)=ε.sinπ.x(n).coscy(n)+β.y(n)mod1

where, *x(n), y(n)* are the chaotic state variables of the map, *ε, β, c* are the control parameters, *n* is the iteration number, and $\mathrm{mod}\left(.\right)$ is the modulo operation symbol. For any strong chaos-based cryptographic method, the chosen chaos should have stable performance which is devoid of periodic regions, low chaotic degree, low complexity, uneven coverage of state-space of its attractors. To performance of the proposed map is assessed through Lyapunov exponents analysis, bifurcation behavior, and phase attractors. The proposed map is also compared with two recently investigated 2-D discrete hyperchaotic map such as 2D-SLIM ($f_{2D-\mathrm{SLIM}}(a,b)$) [30], and 2D-HCM [31] ($f_{2D-\mathrm{HCM}}(r,h)$). These two selected 2D chaotic maps have shown their superiority against many other existing two-dimensional discrete chaotic maps. The 2D-SLIM and HCM chaotic maps have the following Equations (2) and (3), respectively.

(2) f2D-SLIM(a,b)=x(n+1)=sinb.y(n).sin50x(n)y(n+1)=a.1−2.x2(n)sin50y(n)

where (a,b)∈(0,+∞) represent the 2D-SLIM control parameters.

(3) f2D−HCM(r,h)=x(n+1)=sinh.πsin(y(n))y(n+1)=r.sinπ.x(n).y(n)

where *r* and *h* are the 2D-HCM control parameters.

### 3.1. Lyapunov Exponents

**Figure 1:**
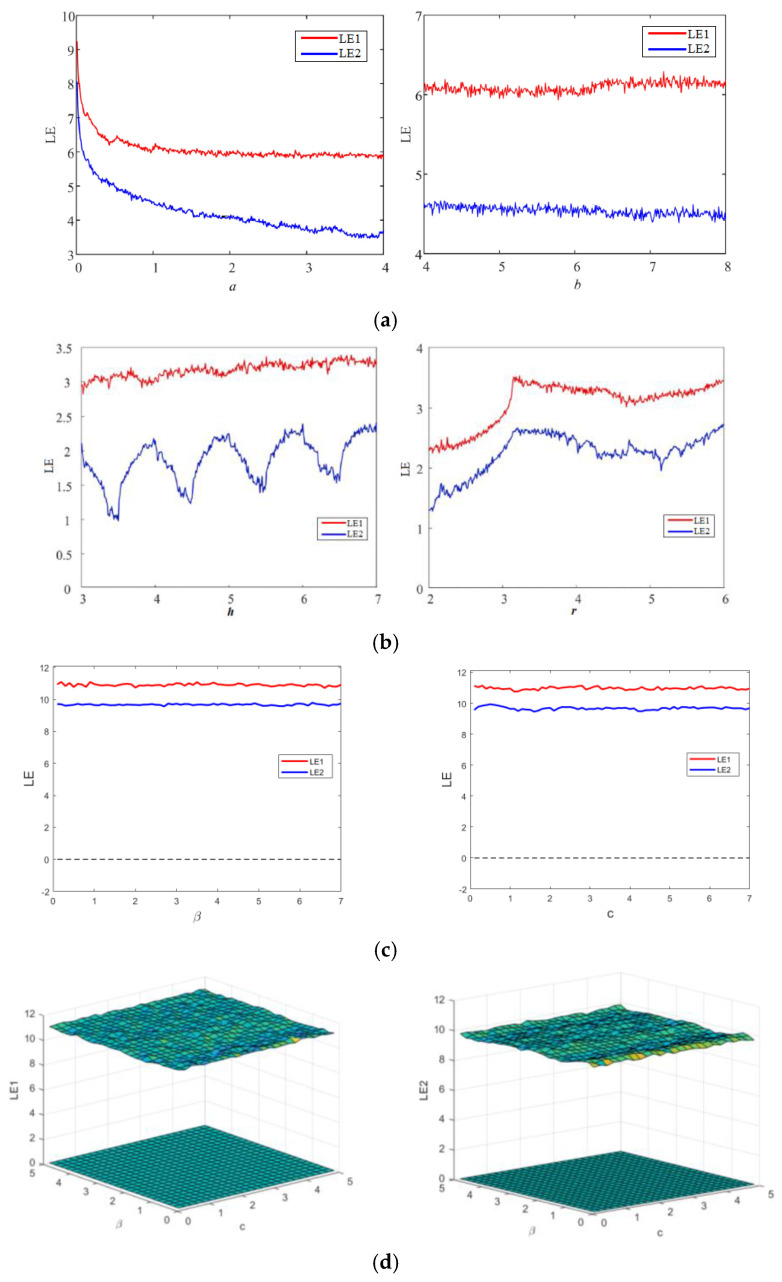
Lyapunov exponents behavior of (**a**) 2D-SLIM, (**b**) 2D-HCM, (**c**,**d**) proposed map.

**Figure 2:**
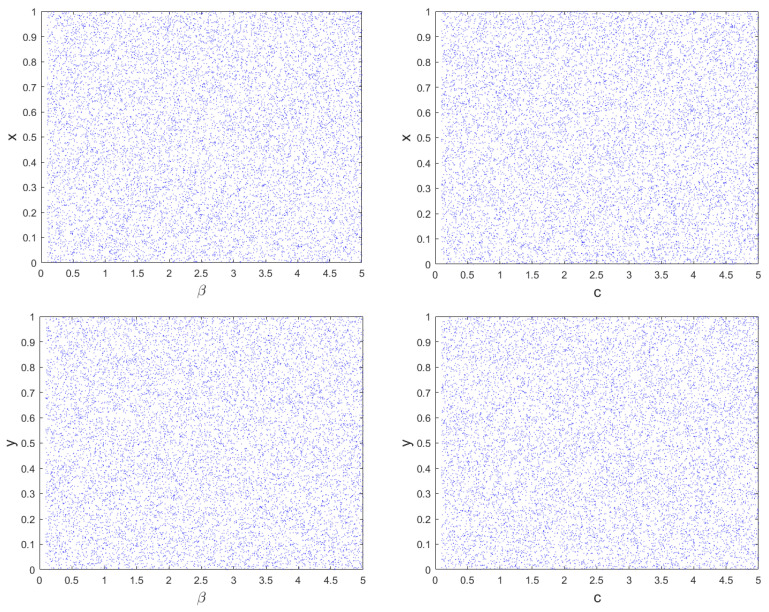
Bifurcation behavior of proposed 2D-MSCM map.

The Lyapunov exponent (LE) is a numerical metric employed to assess the level of chaotic degree in a dynamic system. It is widely recognized as a means to describe the divergence between two trajectories originating from infinitesimally close initial points [8]. The presence of a positive value for the Lyapunov exponent signifies the existence of chaos between two trajectories, as it leads to exponential divergence of the trajectories over time, regardless of their initial state. This characteristic of unpredictability results in a greater exhibition of chaotic behavior in the system when the value of the Lyapunov exponent is higher [32]. The Lyapunov exponent spectrums of the three 2D chaotic maps under examination are shown in Figure 1 for their different control parameters. As seen in the plots in Figure 1, all three chaotic maps have both exponents higher than zero, indicating their hyperchaotic behavior. Figure 1d presents the LE behavior of the proposed map for simultaneous variation in parameters β and c. However, as mentioned, higher value of LE corresponds to the higher chaotic degree, higher sensitivity and complexity of the map. The proposed hyperchaotic map has higher values of both LEs (shown in Figure 1a) compared to the LEs of 2D-SLIM (shown in Figure 1a) and 2D-HCM(shown in Figure 1b) maps for both control parameters. 2D-SLIM and 2D-HCMmaps have proven their better credibility and performance over many chaotic maps of similar dimension. Readers are advised to refer to Refs. [30,31]. Thus, the proposed hyperchaotic map exhibits better and higher chaotic degree and sensitiveness than many recently investigated two-dimensional chaotic maps.

### 3.2. Bifurcation Behaviour

The method of bifurcation analysis is employed to measure the extent of chaotic behavior in nonlinear dynamic systems versus specific system parameter. This analysis depicts the sensitiveness of system to control parameters. A change in these parameters can result in a transition from fixed to chaotic behavior, which is marked by increased randomness in the system outputs. This transition is referred to as a bifurcation. Bifurcation diagrams are used to graphically depict the chaotic behavior of the system [32]. The bifurcation analysis of the proposed hyperchaotic map for control parameters β and c is simulated and behavior is shown in Figure 2. The similar behavior is observed for higher values of both the parameters. Figure 2 evidently displays the non-existence of any discontinuities or periodic windows for both state variables versus both the parameters. Means, the proposed hyperchaotic map has pretty well bifurcation characteristics as needed for strong chaotic systems.

### 3.3. Phase Attractors

**Figure 3:**
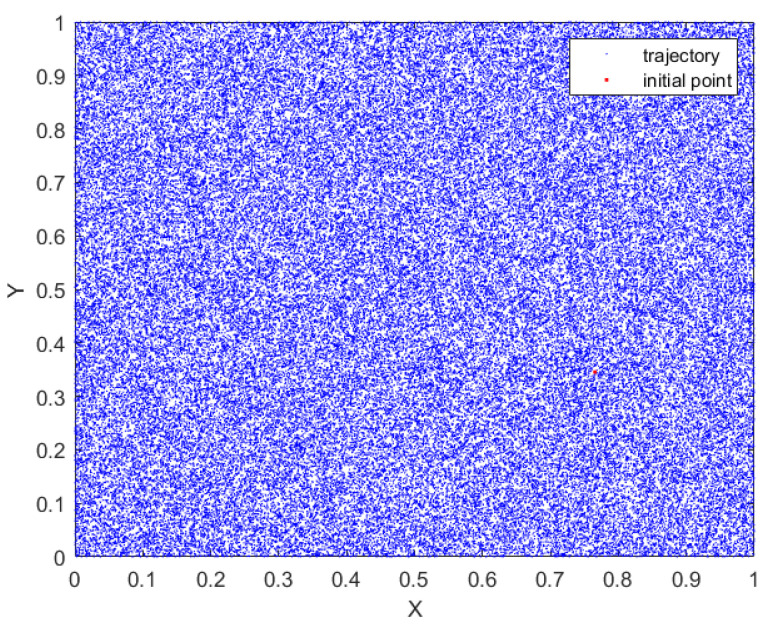
Phase attractor of the proposed 2D-MSCM.

Figure 3 represents the phase diagram to provide an illustration of the coverage of the chaotic attractor of the proposed map. It is crucial to analyze the chaotic attractor in order to gain a comprehensive understanding of the dynamic behavior of chaotic maps [31]. The complex and uniform coverage of complete state-space by the proposed 2D hyperchaotic map indicates its strong and stable performance.

The excellent chaotic behavior of the proposed 2D-MSCM makes it suitable for use in a new chaos and deep learning-based scheme for face tampering detection and self-recovery.

## 4. Proposed Scheme for Color Face Image Authentication and Self-Recovery

Tampered images are often created using a combination of image editing techniques to produce new and different images. The process of image tampering involves substituting a content within a specific area of the original image with other new content [33]. Image tampering can be performed by using different methods including the *copy-move* tampering where a region of an original image is copied and pasted onto another region of the same image [34]. *Cut-and-paste* image tampering where a region of an image is copied and then pasted onto another image [35]. *Image cropping* is the process of removing unwanted parts of an image, such as the background, facial features, or other objects [36]. *Face swapping* is the process of replacing one face with another in an image or video. In recent years, the popularity of *face swapping* has surged, primarily due to advancements in machine learning algorithms [37]. Cyber attackers can use *face swapping* to trick identification or authentication systems and gain unauthorized access [38].

The image tampering attacks discussed above can be applied to color facial images for a variety of purposes, in particular malicious ones. In order to prevent color facial images from being misused by tampering attacks, fragile image watermarking technique can be employed. The latter is designed to detect any minor modification to the authenticated image. This makes it ideal for detecting image tampering attacks [8,18,39].

**Figure 4:**
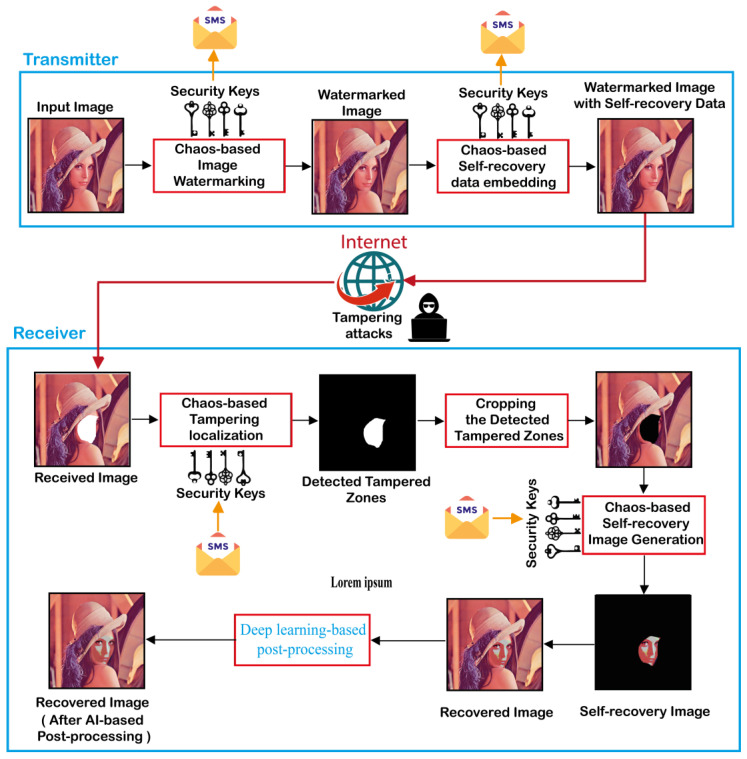
Flowchart of the proposed scheme for color face image authentication and self-recovery.

In this section, a color face image authentication and self-recovery application is introduced in to detect tampered color face images and reconstruct the tampered regions. The proposed application consists of two consecutive phases. The first one is executed at the transmitter side. This phase involves the use of 2D-MSCM for generating the color watermarked image that contains the self-recovery data. Then, the latter is transmitted to the receiver through an unsecured communication channel as the Internet. At the receiver level, the second phase of the proposed application is executed. This phase consists initially in detecting the tampered areas within the received image. Then, removing (cropping) the detected tampered zones from the received image. Next, the recovery image is generated by using a proposed algorithm. Finally, the recovery image is fused with the one containing the cropped areas to generate the recovered image. It should be noted that the proposed application requires the use of symmetrical security keys by both the sender and the receiver. These keys represent the control parameters and the initial values of the 2D-MSCM. Such keys can be transmitted through a reliable communication channel such as the short messaging service (SMS). Therefore, our application can guarantee a high degree of privacy and security. Figure 4 shows the general flowchart of the proposed application and its detailed phases are outlined in the next subsections.

### 4.1. 2D-MSCM-Based Color Image Fragile Watermarking

In order to detect tampered zones within publicly shared images via the Internet, our method consists in including a chaos-based digital signature (watermark) in the original image before it is transmitted over the Internet. For this end, the steps presented in Figure 4 are followed, and detailed specifications of these steps are provided below.

Step 1: This step consists in generating a chaotic-based binary image for use as a secret key to be embedded into the host image. For this, the proposed 2D-MSCM (Equation (1)) is used to produce two chaotic sequences noted X and Y each of size L=N×M where N×M is the host image dimensions. Next, X and Y are binarized as follows:

(4) $$Xb(i)=\left\{\left.\begin{array}{c} 0\;\mathrm{for}\;X(i)<Th1\;\\ 1\;\mathrm{for}\;X(i)\geq Th1 \end{array}\right|\right.\;i=0,1,\ldots ,L$$

(5) $$Yb(j)=\left\{\left.\begin{array}{c} 0\;\mathrm{for}\;Y(j)<Th2\;\\ 1\;\mathrm{for}\;Y(j)\geq Th2 \end{array}\right|\right.\;j=0,1,\ldots ,L$$

Here Th1 and Th2 represent the average values of *X* and *Y* sequences, respectively.

Step 2: Apply the bitwise exclusive OR operator (XOR) operator to *Xb* and *Yb* to generate WB vector as follows:

(6) WB=XOR(Xb,Yb)

The resulting *WB* vector is then reshaped into 2D N×M binary matrix, which represent the watermark image noted *W*.

Step 3: This step consists in splitting the input color image into three RGB channels. Then, one of the latter (i.e., B channel) is selected for *W* image embedding.

**Figure 5:**
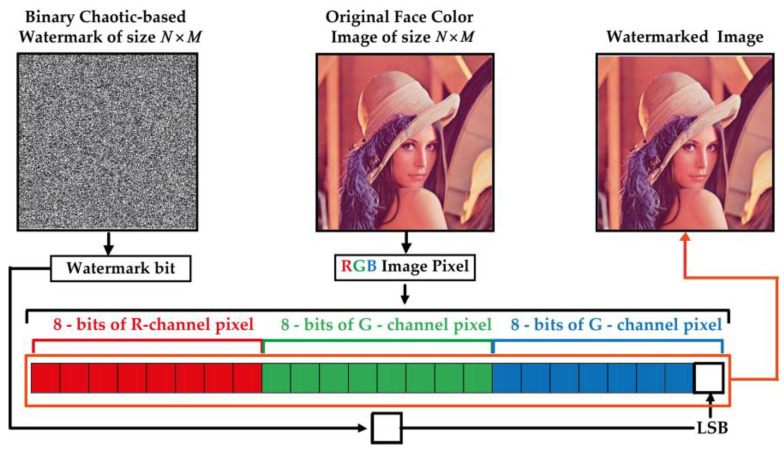
Proposed LSB-based fragile watermarking of RGB color face image.

Step 4: This step consists in substituting the last significant bit (LSB) of the selected channel’s pixels by *W* image bits. Figure 5 shows an illustration of LSB-based color image watermarking process and Algorithm 1 describes this process.
**Algorithm 1.** LSB-based color face image watermarking.**Inputs*****W***: Binary watermark of size N×M generated by the proposed 2D chaotic map
***I***: Input color image of size N×M×3
**Output*****WI***: The watermarked color image
//Splitting the input ***I*** image into three color image channels**1.**Get the red channel (***R***) of the input image (***I***)**2.**Get the green channel (***G***) of the input image (***I***)**3.**Get the blue channel (**B**) of the input image (***I***)**4.**Set the first LSBs of ***B*** to ***W*** bits, which generates B* channel
//Generating the watermarked color image (***WI***)**5.**$WI_{N\times M\times 1}=R$**6.**$WI_{N\times M\times 2}=G$**7.**$WI_{N\times M\times 3}=B*$**8.****Return *WI***

It should be mentioned that the symbol “//” indicates a comment in the algorithms.

### 4.2. Self-Recovery Data Embedding

**Figure 6:**
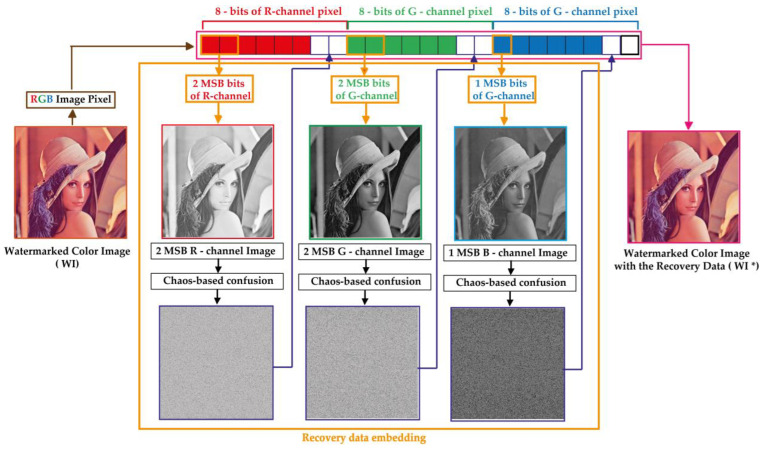
Proposed process for recovery data embedding.

This phase consists in integrating secure data into the watermarked image (*WI*). These data are useful to recovering the significant visual information of the watermarked image from the tampered one. Figure 6 shows the proposed process for embedding the recovery data. The key steps of this process are presented below.

Step 1: This step consists in extracting the most significant two bits of the watermarked image channels (*R* and *G*) to create two binary matrices each of size 2×N×M denoted *Rb* and *Gb*, respectively. Moreover, the MSB of B channel is extracted to produce Bb binary matrix of size N×M.

Step 2: This step is conducted to generate two chaotic sequences noted *X* and *Y* each of size *M* (if *M > N*) by using the proposed 2D-MSCM model. Next, the produced sequences coefficients are rounded to integer values as follows:

(7) $$\begin{array}{l} Xc(i)=\left\lfloor X(i)\times N\right\rfloor \;\mathrm{with}\;i=1,2,\ldots ,N\\ Yc(j)=\left\lfloor Y(j)\times M\right\rfloor \;\mathrm{with}\;j=1,2,\ldots ,M \end{array}$$

where $\left\lfloor .\right\rfloor$ is the floor function.

Step 3: The objective of this step is to confuse the elements of *Rb, Gb* and *Bb* matrices via Algorithm 2.
**Algorithm 2.** Proposed 2D-MSCM based confusion Algorithm.**Inputs:*****Xc***: Confusion vector of size 1×N generated based on 2D-MSCM
***Yc***: Confusion vector of size 1×M generated based on 2D-MSCM
***I***: 2D matrix of size N×M
**Output:*****CI***: The confused version of ***I*** matrix**1.****for *i* = 1 to *N* do****2.**Get $Xc_{i}$ value, which is the *i*-element in *Xc* vector**3.**$I{*}_{i,M}$ ***= CircShift*** ($I_{i,M}$, $Xc_{i}$)//where ***CircShift*** ($I_{i,M}$, $Xc_{i}$) is a left circular shifting function operation, which shifts the elements of *i*-row in *I* matrix by $Xc_{i}$ positions.**4.*****end for*****5.****for *j* = 1 to *M* do****6.** Get $Yc_{j}$ value, which is the *j*-element in *Yc* vector**7.** $I*{*}_{N,j}$ ***= CircShift*** ($I{*}_{N,j}$, $Yc_{i}$)//where ***CircShift*** ($I{*}_{N,j}$, $Yc_{i}$) is a left circular shifting operation, which shifts the elements of *j*-columnin in I* matrix by $Yc_{i}$ positions.**8.*****end for*****9.****for *i* = 1 to *N* do****10.** Get $Xc_{i}$ value, which is the *i*-element in *Xc* vector**11.** $CI_{i,M}$ ***= CircShift*** ($I*{*}_{i,M}$, $Xc_{i}$)**12.*****end for*****13.*****Return CI***

**Figure 7:**
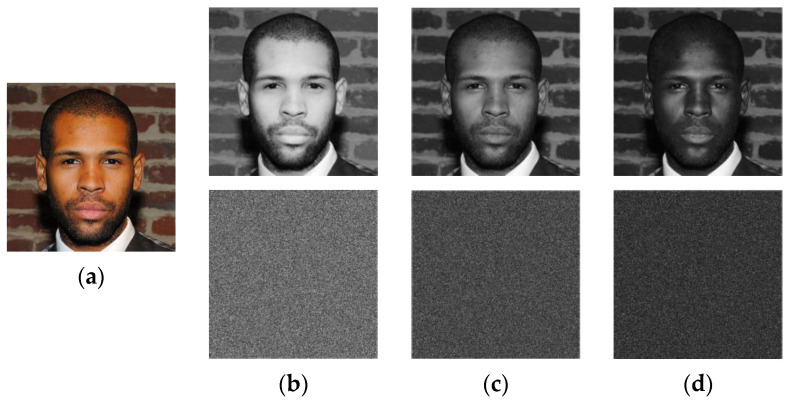
(**a**) Color “Face” image of size 1024 × 1024 and the first row of (**b**–**d**) are *Rb*, *Gb* and *Bb* recovery data obtained from the MSBs of *R*, *G* and *B* channels, respectively, while the second row is their scrambled versions (Rb*, Gb* and Bb*) obtained by using Algorithm 2.

Figure 7 shows *Rb*, *Gb* and *Bb* matrices extracted from “Face” image of size 1024×1024 that is selected from real faces dataset [40]. This dataset that t is publicly available for download contains a collection of 70,000 high-resolution facial images of people from all over the world. The dataset was created by collecting images from the internet and filtering them to remove duplicates and low-quality images. Then, Algorithm 2 is used for scrambling these matrices, which produce the confused versions of the input matrices labeled Rb*, Gb* and Bb*, respectively.

**Figure 8:**
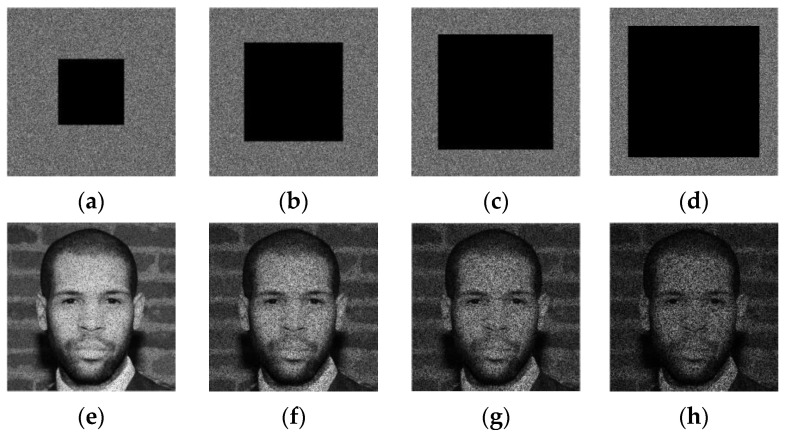
Scrambled R-channel of “Face” image with cropping rate of (**a**) 15.33%, (**b**) 34.44%, (**c**) 61.18%, (**d**) 65.86% and its unscrambled forms: (**e**–**h**) respectively.

From Figure 7, it is observed that the suggested Algorithm 2 entirely masks the visual information of the input image and randomly distributes its visual information whining the whole image area. Therefore, the current stage is designed to strengthen the security level of the proposed scheme and improve its robustness against cropping attacks. Indeed, Figure 8 shows the “Face” image R-channel, which is cropped with different occlusion rates. Then, the reverse operation of the confusion (see Step 3 of Algorithm 5) is applied to the cropped-confused image. This figure shows that the visual information of the entire face remains accessible despite the serious degradation of the confused image by the cropping attack. This important chaos-based property will be exploited in our work to inpainting the tampered face image after removing the tampered regions.

Step 4: This step consists in inserting Rb*, Gb* and Bb* matrices into the LSBs of the watermarked image (WI) channels by using the procedure described in Algorithm 3.
**Algorithm 3.** Proposed Algorithm for the recovery data embedding into the LSBs of the watermarked image channels.**Inputs:**Rb*, Gb*, and Bb*: three binary matrices of size N×M×2, N×M×2 and N×M, respectively
***WI***: Watermarked color image of size N×M×3
**Output:**WI*: Watermarked color image with embedded self-recovery data
//Splitting ***WI*** image into three color image channels**1.**Get the red channel (***RW***) of ***WI*** image**2.**Get the green channel (***GW***) of ***WI*** image**3.**Get the blue channel (***BW***) of ***WI*** image**4.**Get the first layer, noted ***R*1**, of the Rb* matrix**5.**Get the second layer, noted ***R*2**, of the Rb* matrix**6.**Get the first layer, noted ***G*1**, of the Gb* matrix**7.**Get the second layer, noted ***G*2**, of the Gb* matrix**8.**Set the 1-LSBs of the **RW** pixels to ***R1*** bits**9.**Set the 2-LSBs of the **RW** pixels to ***R2*** bits, which generates RW* channel**10.**Set the 1-LSBs of the **GW** pixels to ***G1*** bits**11.**Set the 2-LSBs of the **GW** pixels to ***G2*** bits, which generates GW* channel**12.**Set the 2-LSBs of the **BW** pixels to Bb* bits, which generates BW* channel
//Generating the watermarked color image with the self-recovery data (WI*)**13.**$WI{*}_{N\times M\times 1}=RW*$**14.**$WI{*}_{N\times M\times 2}=GW*$**15.**$WI{*}_{N\times M\times 3}=BW*$**16.**Return WI*

Once Algorithm 3 is executed, the host image can be safely transmitted to a receiver through an insecure communication network.

### 4.3. Blind Detection of the Tampered Areas

The present phase is performed at the receiver side for blindly checking the authentication of the received image and accurately localizing the tampered zones in this image. The next steps are designed for this purpose.

Step 1: In this step, W binary watermark is reproduced by identical manner to that outlined in Step 1 of Section 4.1. It should be noted that at this stage, the receiver must use a symmetric security key to the one used by the transmitter for correctly generate W image.

Step 2: The aim of this step is to accurately localize the tampered regions within the received image by executing the proposed Algorithm 4.
**Algorithm 4.** Proposed algorithm for localizing the tampered areas within the received image.**Inputs:*****W***: Binary watermark of size N×M generated via the proposed 2D-MSCM.
***RI***: Received color image of size N×M×3
**Outputs:*****TZ***: Binary matrix that represents the tampered regions (pixel positions) within the *RI* image
***TZ_NOT***: The logical NOT of TZ matrix
//Splitting ***WI*** image into three color image channels**1.**Get the red channel (***R***) of ***RI*** image**2.**Get the green channel (***G***) of ***RI*** image**3.**Get the blue channel (***B***) of ***RI*** image**4.**Get the first LSB values of ***B*** channel and then save these values in WI* matrix.**5.***T* = XOR(WI*,***W*)**//where the symbol “XOR” denotes the Bit-wise XOR operation between two binary inputs**6.*****TZ* = IMCLOSE(*T*,3)**//where **IMCLOSE(*TZ*,3)** function [41] performs the morphological closing with radius of 3 pixels on ***T*** binary image.**7.*****TZ_NOT* = NOT(*TZ*)**//where **NOT (*TZ*)** operator performs the logical NOT of TZ logical input**8.*****Return TZ, TZ_NOT***

### 4.4. Cropping the Detected Tampred Zones Withing The Received Image

This phase consists in removing the tampered regions for avoid the tamper coincidence problem and then substituting these regions by using the recovery data. To this end, the next steps are involved.

Step 1: This step consists in eliminating the detected tampered regions from the received image as follows:

(8) RI_crop=RI.*TZ_NOT

where “.*” represents the Hadamard product (element–wise multiplication), TZ_NOT is the logical *NOT* of *TZ* binary matrix (see Algorithm 4) and RI_crop represents the RI image after cropping (zeroing) the tampered zones.

### 4.5. Self-Recovery Image Generation

This phase is implemented to generate the self-recovery image that will used for replacing the cropped zones in RI_crop image. To achieve this goal, the next steps are executed.

Step 1: Use Algorithms 5 and 6 for generating the self-recovery image of size N×M×3.
**Algorithm 5.** Proposed algorithm for generating the self-recovery image.**Inputs:*****RI_Crop***: Received image of size N×M×3 with cropped tampered zones
***TZ:*** The logical matrix of the tampered zones
***Xc***: Confusion vector of size 1×N
***Yc***: Confusion vector of size 1×M
**Output:*****SRI***: Generated self-recovery image of size N×M×3**1.**Get the red channel (***R***) of ***RI_Crop*** image**2.**Get the green channel (***G***) of ***RI_Crop*** image**3.**Get the blue channel (***B***) o of ***RI_Crop*** image**4.**Get the first LSB values of ***R*** channel and then save these values in ***R_c1*** matrix**5.**Get the second LSB values of ***R*** channel and then save these values in ***R_c2*** matrix**6.**Get the first LSB values of ***G*** channel and then save these values in ***G_c1*** matrix**7.**Get the second LSB values of ***G*** channel and then save these values in ***G_c2*** matrix**8.**Get the second LSB values of ***B*** channel and then save these values in ***B_c2*** matrix.**9.**Perform the inverse confusion process for ***R_c1*** matrix using ***Inv_confusion*** function described in Algorithm 6 to get ***R1*** matrix**10.**Perform the inverse confusion process for ***R_c2*** matrix using ***Inv_confusion*** function to get ***R2*** matrix**11.**Perform the inverse confusion process for ***G_c1*** matrix using ***Inv_confusion*** function to get ***G1*** matrix**12.**Perform the inverse confusion process for ***G_c2*** matrix using ***Inv_confusion*** function to get ***G2*** matrix**13.**Perform the inverse confusion process for ***B_c2*** matrix using ***Inv_confusion*** function to get ***B2*** matrix
//The following steps are implemented for generating the self-recovery image**14.**SRI_R1 = R1.*TZ//where the symbol “.*” represents the *Hadamard* product operation**15.**SRI_R2 = R2.*TZ**16.**SRI_G1 = G1.*TZ**17.**SRI_G2 = G2.*TZ**18.**SRI_B2 = G2.*TZ**19.**Create an N×M×3 matrix, noted ***SRI***, of zero values represented on 8 bits**20.**Set the 7-MSBs of the first ***SRI*** layer to ***SRI_R1*** binary matrix**21.**Set the 8-MSBs of the first ***SRI*** layer to ***SRI_R2*** binary matrix**22.**Set the 7-MSBs of the second ***SRI*** layer to ***SRI_G1*** binary matrix**23.**Set the 8-MSBs of the second ***SRI*** layer to ***SRI_G2*** binary matrix**24.**Set the 8-MSBs of the third ***SRI*** layer to ***SRI_B2*** binary matrix**25.****Return *SRI***

**Algorithm 6.** Proposed inverse confusion algorithm (***Inv_Confusion***) based on 2D-MSCM.
**Inputs:**
***Xc***: Confusion vector of size 1×N generated based on 2D-MSCM
***Yc***: Confusion vector of size 1×M generated based on 2D-MSCM
***CI***:Confused 2D matrix of size N×M

**Output:**
***I***: The inverse confused version of ***CI*** matrix
**1.**

**for i = 1 to *N* do**

**2.**
 Get $Xc_{i}$ value, which is the *i*-element in *Xc* vector
**3.**
$CI{*}_{i,M}$***= CircShift*** ($CI_{i,M}$, $-Xc_{i}$) 
**4.**

***end for***

**5.**

**for *j* = 1 to *M* do**

**6.**
 Get $Yc_{j}$ value, which is the *j*-element in *Yc* vector
**7.**
 $CI*{*}_{N,j}$ ***= CircShift*** ($CI{*}_{N,j}$,$-Yc_{j}$) 
**8.**

***end for***

**9.**

**for *i* = 1 to *N* do**

**10.**
 Get $Xc_{i}$ value, which is the *i*-element in *Xc* vector
**11.**
 $I_{i,M}$ ***= CircShift*** ($CI*{*}_{i,M}$,$-Xc_{i}$) 
**12.**

***end for***

**13.**

***Return I***


Step 2: This step consists in producing the recovered image (Rec) as follows:

(9) Rec=RI_Crop+SRI

where *RI_Crop* represents the *RI* after cropping the tampered zones and SRI is the self-recovered image. It should be noted that each RGB pixel in the *SRI* image is generated from only 5 bits, while 19 bits of this RGB pixel still missing (Figure 6). Therefore, a post-processing phase is necessary to improve the quality of *Rec* image. It is also noteworthy that Algorithms 5 and 6 are considered as inpainting tools in our framework, as they allow filling the cropped areas in *Rec* image.

### 4.6. Deep Learning-Based Post-Processing of the Recovered Image

The current phase is designed to improve the visual quality of the recovered image (Rec). To this end, two consecutive post-processing steps are undertaken. The first one, consists in filling the zero-valued pixels in Rec image. To this end, zero values are replaced by neighboring Rec image values. The second step involves the use of a pre-trained deep learning models, which are used to refine and improve the visual quality of Rec image.

For improving the visual quality of the recovered image, it goes through a post-processing stage where deep learning models are deployed. Indeed, the pretrained CodeFormer [16] model is initially used with its default parameters [42] to improve the visual quality of the recovered image. This model was originally designed for blind restoration of natural face images from heavily degraded ones. To improve the colorization of the output image of CodeFormer model, we use the DeOldify [17] model. This model is an open source fully automatic colorization method created by Jason Antic. The full code of this model is available in [17]. It is an End-to-End CNN-based model that yields impressive image and video colorization results. It is important to note that the proposed scheme currently uses the default parameters of the involved deep learning models. However, in the future, more focus can be devoted to optimizing the model parameters to improve the proposed scheme performance.

**Figure 9:**
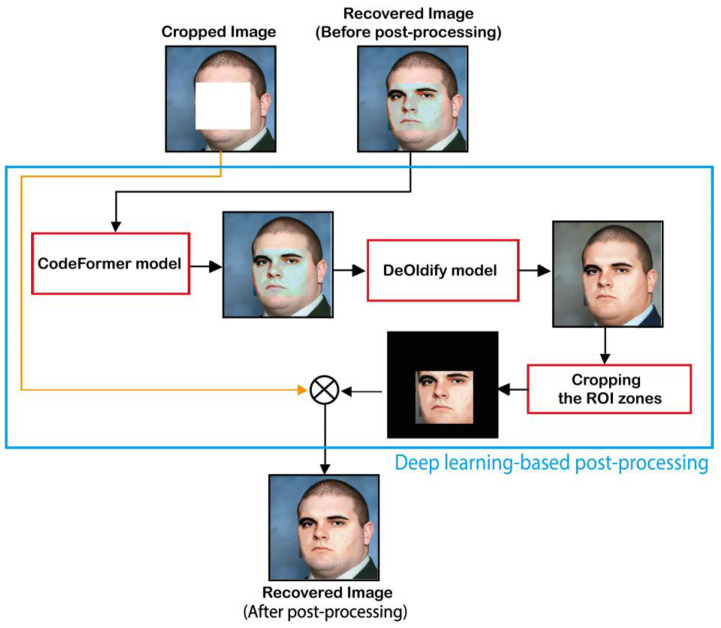
Deep learning-based post-processing of the recovered image.

Figure 9 shows the deep learning-based post-processing phase involved in our scheme. This phase image is accomplished as follows:

Step 1: Use the CodeFormer model to improve the visual quality of Rec image. Then, the output image labeled Rec1 image is used as input of DeOldify model for improving the colorization of the facial image. Finally, the output image of DeOldify is labeled Rec2.

Step 2: This step consists of cropping the ROI zones, which represent the enhanced self-recovery image as follows:

(10) Rec_crop= Rec2 .*TZ

Step 3: In this step, the final recovered image, denoted by Rec_Final, is generated by merging Rec_crop with RI_crop images as follows:

(11) Rec_Final=Rec_crop+RI_crop

After outlining the functionality of the proposed application, the following section presents the simulation results achieved by using this application.

## 5. Simulation Results

This section includes the simulation findings that demonstrate the good performance of the method. To this end, this section focuses on demonstrating four main aspects related to the proposed scheme, namely (i) the imperceptibility of the input image after including the watermark and self-recovery data, (ii) the accuracy of detecting the tampered areas, (iii) the security level, and (iv) the quality of the self-recovered image. The experiments are performed using Matlab software implemented on a personal laptop with RAM of 16 GB and CPU 2.1 GHz.

### 5.1. Evaluation of Imperceptibility Performance

The proposed scheme involves embedding the watermark and the self-recovery data into the input image that leads to certain degradation of this image. Since the proposed scheme inserts the watermark and the self-recovery data into the 2 LSBs of the input image channels, it is considered as fragile watermarking scheme. Such schemes typically ensure a good quality of the watermarked image. To verify this assumption, the present analysis is conducted.

To quantify the effect of the watermark and the recovery data embedding on the quality of the host image, PSNR and SSIM (structural similarity index) are used. These criteria are defined below.

(12) $$PSNR=10\log_{10}\frac{P^{2}}{\frac{1}{NM}\sum_{x=0}^{N-1}\sum_{y=0}^{M-1}\left[I(x,y)-IW(x,y)\right]}$$

where *P* is the peak value of the host image *I(x,y)* and *IW(x,y)* is the resulting image after embedding the watermark and self-recovery data.

(13) $$\mathrm{SSIM}=\frac{\left(2\mu_{f}\mu_{f^{\prime }}+c_{1})(2\sigma_{f,f^{\prime }}+c_{2}\right)}{\left(\mu_{f}^{2}+\mu_{f^{\prime }}^{2}+c_{1})(\sigma_{f}^{2}+\sigma_{f^{\prime }}^{2}+c_{2}\right)}$$

where $\sigma_{f,f^{\prime }}$ is the covariance between the cover image and its modified version, ($\mu_{f}$, $\sigma_{f}^{2}$) are mean and variance of the cover image, respectively, and ($\mu_{f^{\prime }}$, $\sigma_{f^{\prime }}^{2}$) are those of the modified image, and (*c*_1_, *c*_2_) are constant values added to prevent division by zeros.

The mean squared error (MSE) is a common metric used to quantify the difference between the pixel values of the original image and its modified version. It is calculated as follows:

(14) $$MSE=\frac{1}{NM}\sum_{x=0}^{N-1}\sum_{y=0}^{M-1}\left[I(x,y)-I^{\prime }(x,y)\right]$$

where I is the original image of size N×M and $I^{\prime }$ its modified version.

It should be noted that the degree of degradation in the pixel values between the original image and it tampered version can be quantified by using common metrics including the MSE, PSNR and SSIM [3,43,44].

**Figure 10:**
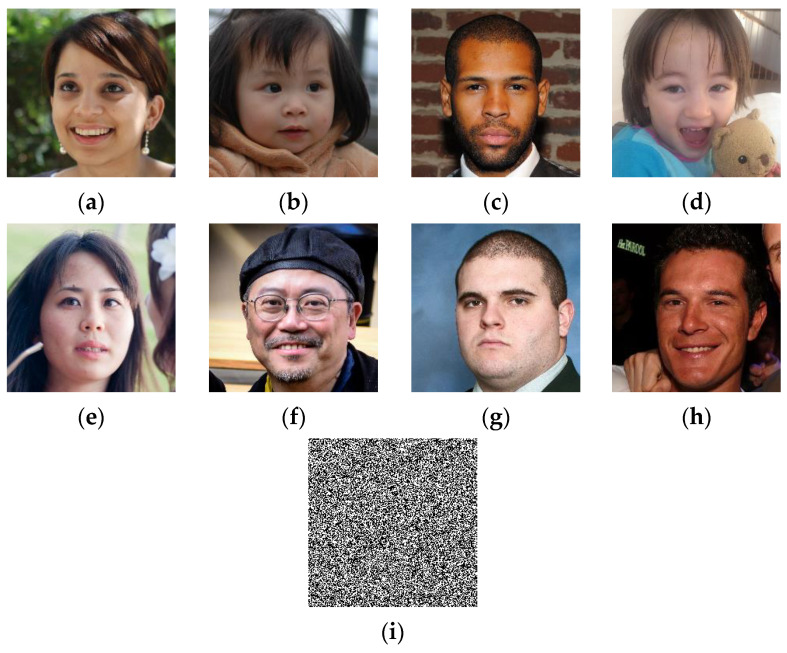
(**a**–**h**) Set of 1024 × 1024 and (**i**) with a binary watermark image generated by 2D-MSCM.

For performing the current test, we use a set of color face images of size 1024×1024, which are randomly selected from [40] dataset. Then, a binary watermark (*W*) of size 1024×1024 is generated based on 2D-MSCM where the parameters of the latter are set to $\left(e,c,b,X_{0},Y_{0})=(100,5,5,0.7654,0.3456\right)$. The recovery data are then constructed from each image and integrated into the host images using the proposed method. Figure 10 shows test images with *W* image generated by 2D-MSCM. Figure 3 shows the test images with the corresponding PSNR and SSIM values after incorporating the watermark image and the recovery data.

**Figure 11:**
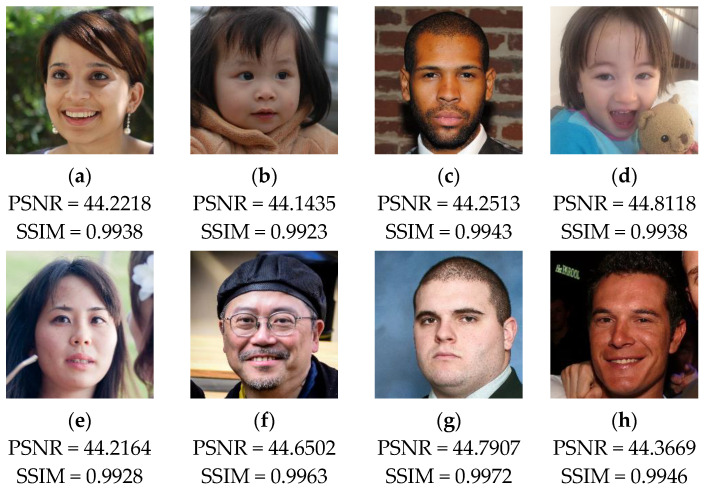
(**a**–**h**) Test images following the embedding of the watermark and the recovery data with the corresponding PSNR and SSIM values.

The test results provided in Figure 11 show that the embedding of the watermark and the recovery data slightly reduces the visual quality of the host images. In fact, we can notice that the values of PSNR and SSIM remain higher than 44 dB and 0.99, respectively, for all the test images. Therefore, the proposed method provides good performance in terms of imperceptibility property. This result can be interpreted by the fact that our method incorporates the data into two LSBs of each pixel, which lead to low degradation of the input image’s visual quality.

### 5.2. Evaluation of Tampering Detection Rate Performance

This section contains a set of experiments that test the accuracy of the proposed scheme in detecting the tampered regions within the watermarked image. To evaluate the accuracy of proposed method for tampering detection, the following criteria are used:

(15) $$\mathrm{Accuracy}=\frac{TP+TN}{TP+TN+FP+FN}$$

(16) $$\mathrm{Recall}=\frac{TP}{TP+FN}$$

(17) $$\mathrm{Precision}=\frac{TP}{TP+FP}$$

where *TP* is the true positive value, which indicates the number of the correctly detected pixels within the tampered zone. *FN* is the false negative; i.e., the number of undetected pixels within the tampered area. *FP* represents the number of the pixels that are incorrectly detected in the non-altered zone. *TN* represents the true negative; i.e., is the number of undetected pixels within the un-tampered area.

**Figure 12:**
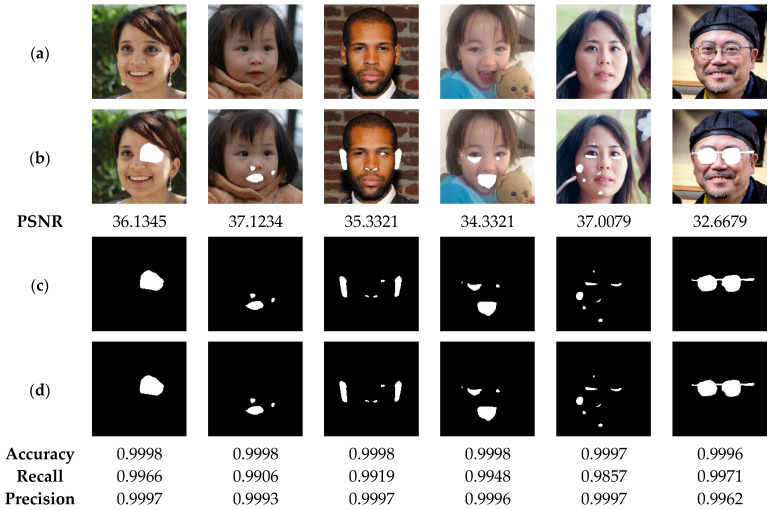
(**a**) Watermarked test images of size 1024 × 1024. (**b**) Images attacked by cropping with the corresponding PSNR values. (**c**) Ground truth binary masks. (**d**) Detected tampered zones with the corresponding accuracy, recall and precision values.

To perform the present analysis, the watermarked images shown in Figure 12 are used. Next, the latter are manipulated by different types of tampering attacks, namely: irregular shape cropping with different rates, copy-move, and face swab attack. The cropping attack consists in removing certain areas of an input image. The removed areas are then replaced by 255 grayscale value. The copy-move attack consists in copying a part of the image and duplicating the copied part in another location of the same image. The face swap attack involves replacing the face in an image with another face imported from another image. Clearly, this attack type is one of the most serious attacks, which can lead to harmful consequences for victims of such attack. The image cropping and copy-move attacks are performed with the “Paint” tool of the Windows Operating System (WOS). This tool is widely used by WOS users for manipulating the digital images. The face substitution attack is performed via the deep learning-based [45] platform. Matlab software is then used to copy the face region from the output image of [45] platform into the watermarked image. For each tampered image, the tampered area is labelled as the ground truth. For each tampered image, the tampered area is labeled as ground truth. Then, the proposed method is used to detect the tampered areas within the tampered image. Finally, the criteria given by Equations (15)–(17) are used for evaluating the performance of our method.

**Figure 13:**
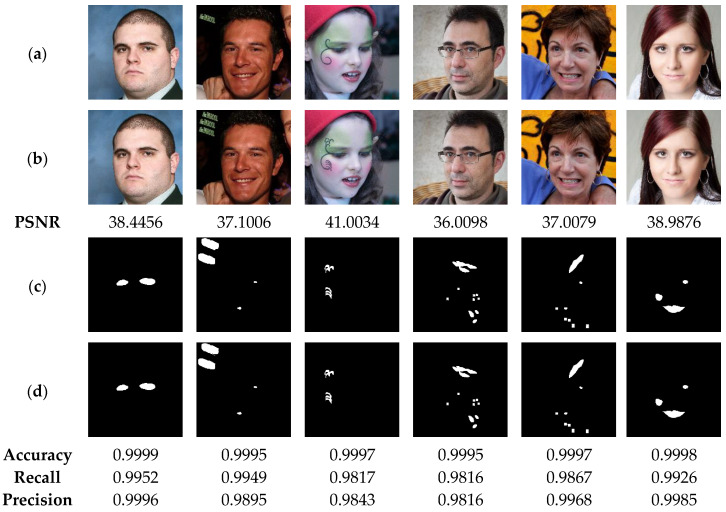
(**a**) Watermarked test images of size 1024 × 1024. (**b**) Copy-move attacked images with the corresponding PSNR values. (**c**) Ground truth binary masks. (**d**) Detected tampered zones with the corresponding accuracy, recall and precision values.

**Figure 14:**
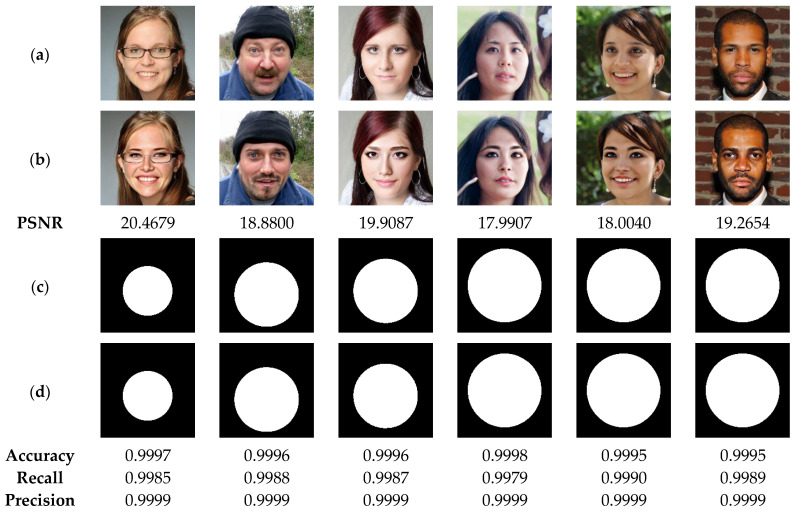
(**a**) Watermarked test images of size 1024 × 1024. (**b**) Attacked images by face swapping with the corresponding PSNR values. (**c**) Ground truth binary masks. (**d**) Detected tampered zones with the corresponding accuracy, recall and precision values.

Figure 12 illustrates the performance of our method in detecting the deleted areas of irregular geometry. The test results indicate the high efficiency of our method in detecting the tampered areas with accuracy, precision and recall higher than 99%. This high performance can be explained by the fact that our method is designed to detect the tampering in each pixel of the authenticated image. In addition, Figure 13 show that our method is able to detect tampered regions by copy-move attack with high accuracy, greater than 99%, which provides a clear indication of the effectiveness of our method in detecting the copied-moved regions, which are extremely difficult to be sensed by the human vision system. Furthermore, the results presented in Figure 14 indicate that the proposed scheme can detect the swapped faces with more than 99% of accuracy. Meanwhile, when using the human vision system, it is very difficult, if not almost impossible, to detect the fake faces. It is therefore advisable to integrate the proposed technology into camera-based smart devices in order to avoid face-swapping attacks that can negatively affect the privacy of victims.

### 5.3. Evaluation of Image Recovery Performance

**Figure 15:**
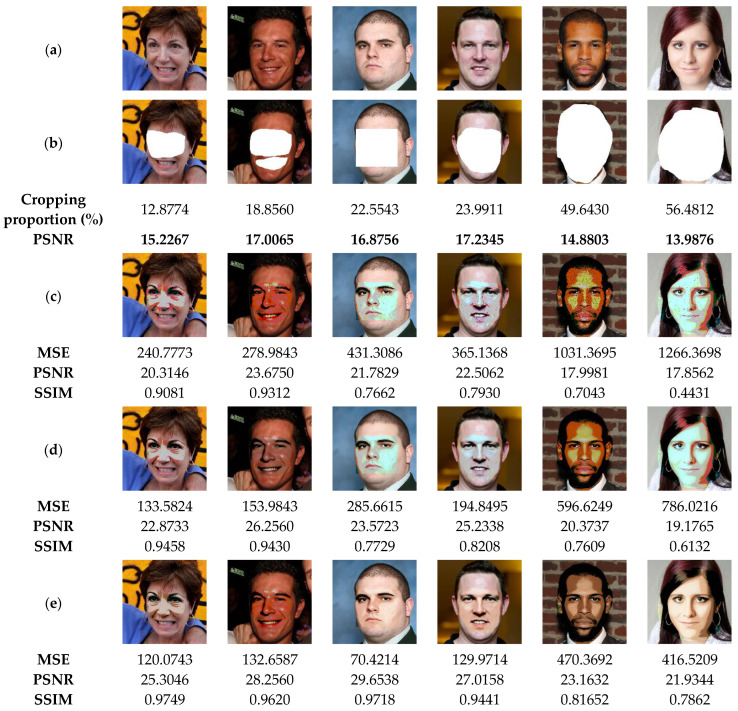
(**a**) Watermarked test images of size 1024 × 1024. (**b**) Attacked images by face removing with the corresponding cropping proportions and PSNR values. (**c**) Recovered images by the proposed method before the post-processing. (**d**,**e**) Recovered images after applying the CodeFormer and DeOldify models, respectively, with the corresponding MSE, PSNR and SSIM values.

The suggested method not only detects the tampered areas in color images with high accuracy, but also recovers the original information of the tampered regions. For this end, the current section is devoted to evaluating the performance of our method in recovering the original data of the tampered areas, especially those of significant proportions. To perform the current analysis, the watermarked images (Figure 15a) are attacked by removing regions of these images by of various proportions as shown in Figure 15b, which causes a strong degradation of the facial shape. After detecting the tampered areas, our method is used to recover the lost data of such areas. Figure 15c shows the recovered images through our method before applying the deep-learning based post-processing. This figure indicates that the proposed method can recover the important visual pattern of the deleted areas from the face image. This is a clear indication of the effectiveness of the proposed method. However, the visual quality of the recovered image remains unsatisfactory. By using the CodeFormer model (Figure 15d), we can notice that the quality of all the recovered images is improved. This result indicates that the incorporation of this deep-learning model provides a significant benefit in improving the quality of the recovered images. To further improve the colorization of the recovered images, DeOldify model is used. From Figure 15e, it can be seen that the use of this model further improves the quality of the recovered image by enhancing the face image colorization. From the current analysis, it is clear that the use of deep-learning models significantly improves the quality of the recovered images. Accordingly, further investigation is needed in future work to test and compare other deep-learning models in image tampering detection and self-recovery.

**Figure 16:**
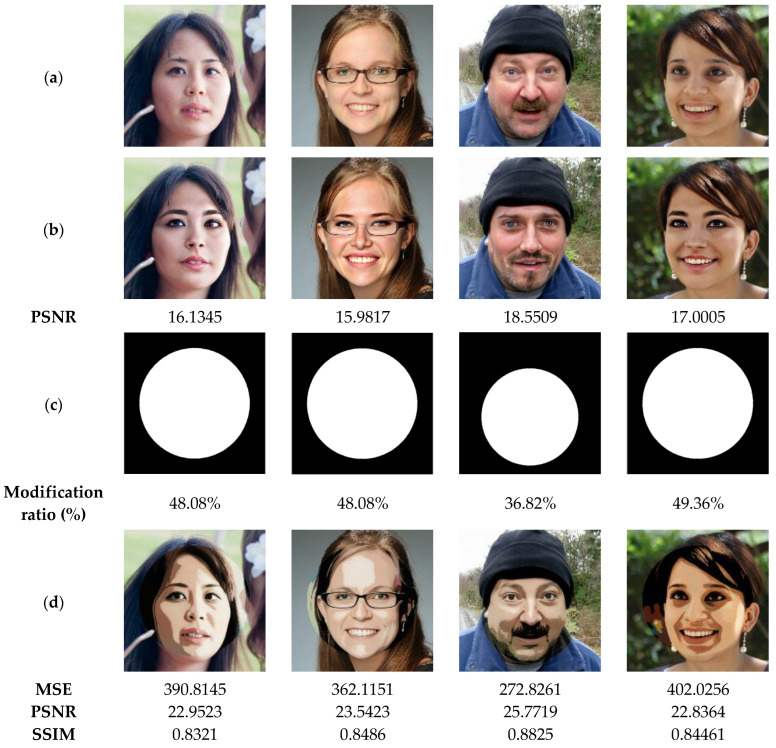
(**a**) Watermarked test images of size 1024 × 1024. (**b**) Attacked images by face with the corresponding PSNR values. (**c**) Ground truth binary masks. (**d**) Detected tampered zones with the corresponding accuracy, recall and precision values.

In the next test, the performance of our method is tested to recover original images from attacked ones via swap-face attack of high proportions. The results illustrated in Figure 16 show the similarity between the recovered images and the original images is acceptable (SSIM > 0.82), even if the tampering rates of the exchanged faces are important (>48%). Therefore, our method can be considered as a useful application for detecting and restoring tampered areas within the significantly distorted images.

**Figure 17:**
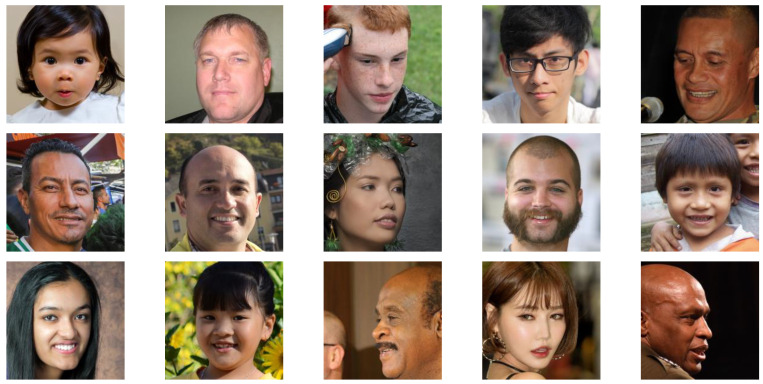
Set of color face images with resolution 512 × 512 selected from the dataset [46].

In the next test, we randomly selected 5000 color face images from the dataset [46] to further validate the performance of our scheme. The dataset [46] consists of 52,000 PNG images of faces at 512 × 512 resolution. The images cover a wide range of ages, ethnicities, and image backgrounds, as well as accessories such as eyeglasses, sunglasses, and hats. The images were collected from Flickr and automatically aligned and cropped. Figure 17 shows a set of color face images used in the present test.

**Figure 18:**
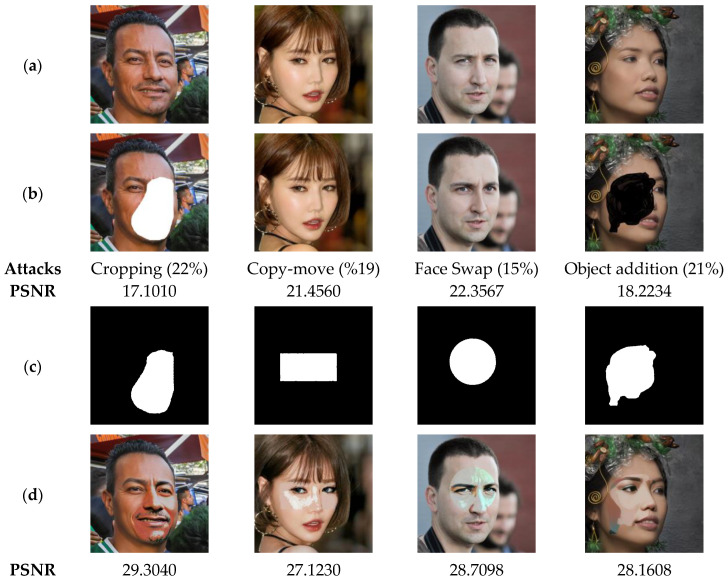
(**a**) Watermarked test images. (**b**) Attacked images by various attacks with different proportions and PSNR values. (**c**) Detected tampered zones. (**d**) Recovered color face images by our method with the corresponding PSNR values.

In the current test, the selected images are subjected to various attacks mentioned in Table 2. The performed attacks are controlled to affect the color faces by a proportion of up to 25%. Next, the proposed method is used to recover the face image from the attacked ones, and the average of the PSNR values corresponding to all recovered images is reported in Table 2. A set of the original test images, their attacked versions, and recovered ones are presented in Figure 18. It should be mentioned that the images in the dataset [46] do not belong to the test images used in the training phase of the proposed scheme.

The results in Figure 18 and Table 3 show that the PSNR values of recovered images decrease as the attacked surface area increases. However, the quality of recovered images remains good (PSNR > 26). These results confirm that our method is valid for ensuring the authenticity of color face images belonging to different races.

**Figure 19:**
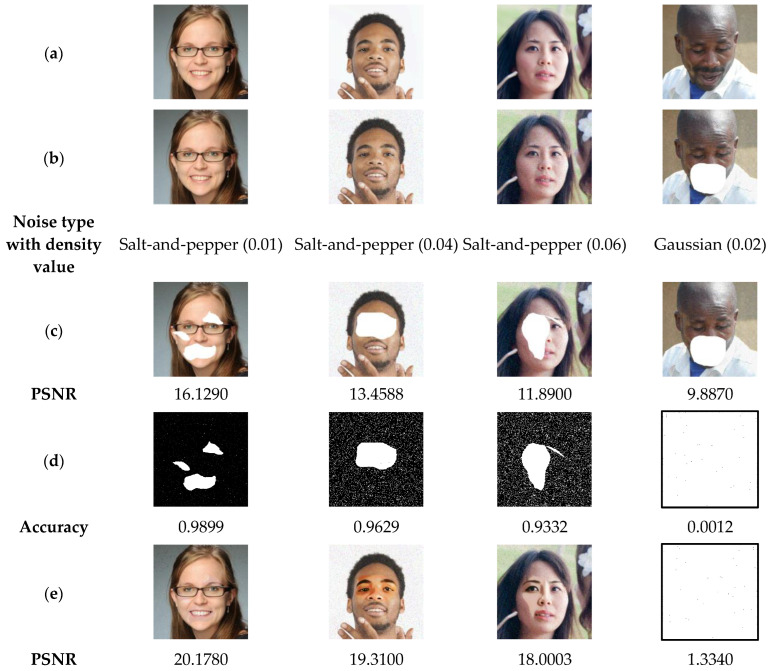
(**a**) Watermarked test images of size 512 × 512. (**b**) Attacked-watermarked images by “Salt-and-pepper” and “Gaussian” noise with various densities. (**c**) Noised-cropped images with the corresponding PSNR values. (**d**) Detected tampered zones with the corresponding accuracy, and (**e**) the recovered color face images by our method and their PSNR values.

Noise can be produced when transmitting images over communication channels. This noise can be caused by the physical characteristics of the channel, such as the distance between the transmitter and receiver and the bandwidth of the channel [47]. In the following test, the proposed method is evaluated against two types of noise: “Salt-and-pepper” and “Gaussian”, with different densities. Figure 13 shows the results of the current test, which show that our proposed scheme can accurately detect the tampered regions in the watermarked-noised image by salt-and-pepper noise with densities up to 0.06. The recovered images in Figure 19 also have acceptable quality (PSNR > 28). Therefore, our method can be considered robust against salt-and-pepper noise. However, if the watermarked image is attacked by Gaussian noise, our method cannot detect the tampered regions and therefore cannot recover the color face image. Thus, the proposed method is not robust to Gaussian noise. This limitation presents an interesting research opportunity for future work.

Salt-and-pepper noise is a type of noise that causes random pixels in a color image to be set to either 0 or 255 values. This type of noise is often caused by bit errors in digital transmission. Gaussian noise, on the other hand, is a type of noise that is characterized by a normal distribution and affects all the image pixels. It is often caused by random fluctuations in the environment, such as thermal noise. Therefore, the current test results can be explained by the fact that the proposed method is robust to salt-and-pepper noise because it is able to identify and remove these pixels from the color face image. However, it is not robust to Gaussian noise because it is unable to distinguish between Gaussian noise values and the original image pixel values. Note that the nearly blank image in the last column of row (d) in Figure 19 indicates that almost all pixels (>99%) in the Gaussian noised image are considered tampered and are therefore replaced by 1 s in the corresponding binary mask image. Also, the nearly blank image in the last column of row (e) in Figure 19 indicates that the recovered color face image pixels are not correctly retrieved and are therefore replaced by 255 s, resulting in a nearly blank image.

It is important to mention that our method inserts the watermark and recovery data into the least significant 2 bits (LSBs) of each color face image pixel. The watermark data is embedded in the 1 LSB of one secret image channel, which makes it difficult for malicious attacks to forge or remove the watermark without removing the least significant bit of the entire color face image. To make the proposed method more robust against watermark data deletion attacks, the watermark data can be inserted into one of the 2 -LSBs of a secret color face image channel, so that deleting the watermark data is expected when deleting all the 2-LSBs of the three-color image channels. Additionally, the proposed system can be adapted to use multiple chaotic watermarks, so that if one watermark set is corrupted or lost, the others can still be used. In addition, the proposed system incorporates a pseudo-randomly distributed chaotic watermark (Algorithm 1) throughout the image area, making it difficult to falsify or remove the watermark without seriously damaging the watermarked color face image.

To make our scheme more robust against malicious attempts to forge or remove the recovery data, we can use multiple sets of redundant recovery data embedded in the 3-LSBs of the image channels. This would make it more difficult for an attacker to remove all of the recovery data without seriously damaging the watermarked color face image. However, embedding the recovery data in the 3—LSBs of the watermarked image channels can reduce the image quality by introducing noise-like artifacts. This is especially noticeable in areas of the image with a lot of detail. In future work, more attention should be paid to developing methods that can achieve good watermarked image quality while incorporating a large amount of recovery data.

It is important to mention that the proposed scheme is of low robustness to lossy compression attacks. This is because it embeds the watermark and recovery data in the 2-LSBs of the image. The latter are the least important bits of an image, and they are often discarded by lossy compression algorithms [48] in order to reduce the size of the image. To overcome this limitation, a lossless image compression algorithm can be used before transmitting the watermarked color face image. Lossless compression algorithms do not remove any data from the image, so the watermark and recovery data would be preserved.

The previous discussion shows that the proposed scheme is a promising approach to guaranteeing the authenticity and integrity of color face images. To further demonstrate the security performance of the proposed scheme, the following evaluation is presented.

### 5.4. Evaluation of Security Performance

This section presents the experiment finding that demonstrate the security performance of the suggested method. For this purpose, two critical aspects related to the security requirements are investigated, namely the key space and the sensitivity of the proposed scheme to its security keys.

#### 5.4.1. Key Space Analysis

In this subsection, the key space of our scheme is calculated to show the ability of this scheme to withstand brute force attacks. The proposed scheme security key is composed of two parts: the first one is used during the watermark generation phase, and the second one is created when embedding the recovery data. In each phase, the initial values and the control parameters of 2D-MSCM are used as components of the security key denoted $KEY=\left\{\left\{Key1\right\},\left\{Key2\right\}\right\}=\left\{\left\{\varepsilon ,\beta ,c,x_{0},y_{0}\right\},\left\{\varepsilon *,\beta *,c*,x_{0}*,y_{0}*\right\}\right\}$ that consists of 10 real numbers. Given the limited precision of the computer to the order of $10^{-15}$, the KEY space becomes approximately ${\left(10^{15}\right)}^{10}=10^{150}≃2^{494}$. This size far exceeds the recommended minimum size of $2^{100}$ [49]. Therefore, the proposed system is capable of withstanding brute force attacks of modern computers.

#### 5.4.2. Key Sensitivity Analysis

**Figure 20:**
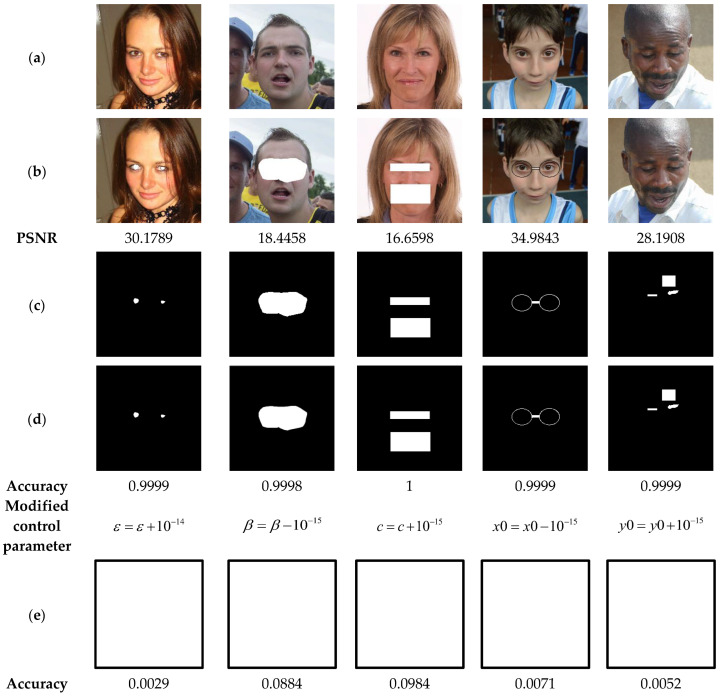
(**a**) Watermarked test images of size 1024 × 1024. (**b**) Tampered images by cropping attacks with the corresponding PSNR values. (**c**) Ground truth binary masks of the tampered areas. (**d**,**e**) Detected tampered zones with the corresponding accuracy when using the correct security key and incorrect one, respectively.

The present analysis evaluates the sensitivity of our scheme to the used security keys. For this, in the watermarking phase, the security key marked $Key1=\left\{\varepsilon ,\beta ,c,x_{0},y_{0}\right\}=\left\{100,5,5,0.7654,0.3456\right\}$ is used in the suggested method to generate the binary watermark that is embedded into the test images shown in Figure 20a. Then, the watermarked images are attacked by various attacks (Figure 20b). Subsequently, the proposed method is used to detect the attacked areas within the watermarked image in two scenarios. The first one consists in the use of the correct security key (*Key1*) during the tampering detection phase (Figure 20d), and the scenario involves using an incorrect security key obtained through the modification of only one element of *Key1* by a variation of the order $\pm 10^{-15}$ (Figure 20e). Finally, the proposed method is employed to locate the tampered areas using the correct security key and the wrong one, respectively. Note that the white color in Figure 20e indicates that the proposed scheme is highly sensitive to any slight variations in the security key, and therefore any tampered zone in the input image is localized.

**Figure 21:**
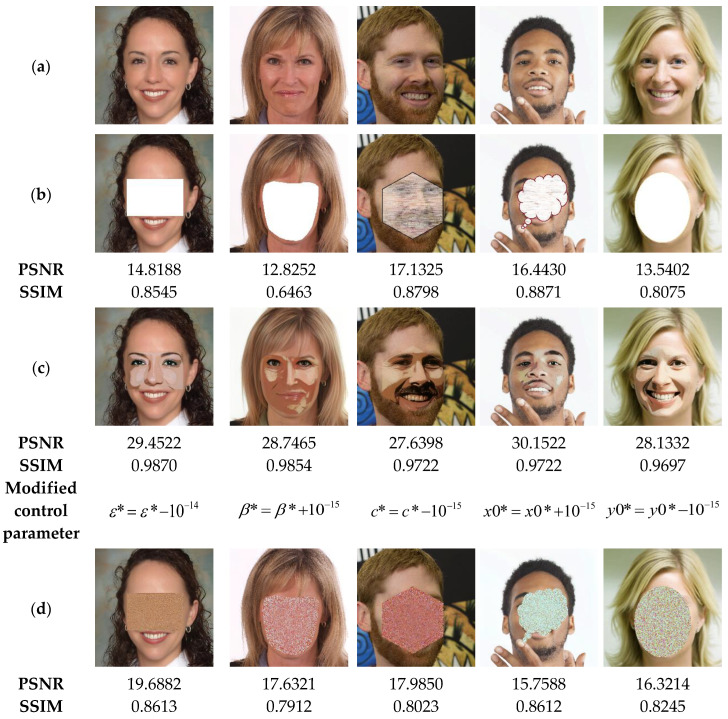
(**a**) Watermarked test images of size 1024 × 1024. (**b**) Tampered images. (**c**,**d**) Recovered images with the corresponding PSNR and SSIM values when using the correct security key and incorrect ones, respectively.

The results of the current tests are shown in Figure 21. This figure illustrates on the one hand that when the correct security key is used, the tampered areas are detected with high accuracy (>99%). On the other hand, when an incorrect security key is employed, our scheme is unable to detect the tampered areas since its detection accuracy is close to 0%. These outcomes are a clear indication regarding the high sensitivity of our system to its security keys, which proves the safety level and reliability of the suggested scheme. To support this finding, the test shown in Figure 21 is performed. In this test, the watermarked images are subjected to tampering attacks. Then, in the image recovery phase, we use both the correct security key, denoted *Key*2 with $Key2=\left\{\varepsilon *,\beta *,c*,x_{0}*,y_{0}*\right\}=\left\{100,5,5,0.7654,0.3456\right\}$ and incorrect keys. The results of the current test analysis indicate that using the correct security *Key*2 effectively recovers the original information of the tainted areas (PSNR > 27). In contrast, when a single parameter of *Key*2 is slightly changed by $\pm 10^{-15}$ during the data recovery process, the suggested scheme is unable to recover any useful information of the tampered zones. Therefore, the use of the proposed system guarantees a high level of security.

Note that the blank binary mask images in row (e) of Figure 20 indicate that almost all pixels (>99%) in the attacked watermarked images are considered tampered and are therefore replaced by 1 s in the corresponding mask images.

### 5.5. Comparison with Similar Work

**Figure 22:**
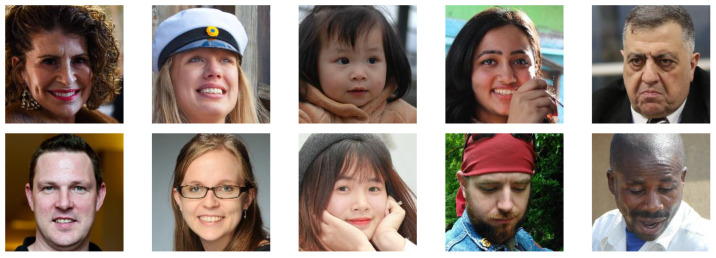
Set from the test images selected from the dataset [40] and used in the comparative analysis.

This section evaluate the performance of our scheme and in comparison recent color images tamper detection and self-recovery schemes presented in [3,4,8,25]. To perform this comparison, 100 test images (Figure 22) are arbitrarily selected from the dataset [40]. These images are then subjected to irregular cropping form attack with various proportions up to 50% (Table 2). Next, the compared schemes are used to detect the tampered areas in the test images. Finally, the average value of the precision metric is computed for each method and reported in Table 2. The results presented in this table demonstrate the superiority of our scheme over the compared ones for the detection of tampered zones with irregular shapes. This superiority can be is explained by the fact that our method is pixel-based, contrary the other schemes are block-based. The latter increase the rate of the false positive, which leads to decreased accuracy of the such schemes, especially in irregularly shaped tampering detection.

To further demonstrate the high-precision of the proposed method in detecting the image tampering, the test images are attacked by copy-and-move attacks with various proportions of the irregular copied-moved zones. Then, the compared schemes are used for detecting the tampering. The average precision values achieved for each scheme are given in Table 3. The latter indicates that the proposed scheme outperforms the compared schemes, supporting the superiority of the proposed scheme over the state-of-the-art schemes in terms of precision of tampering detection.

The present comparative analysis also focuses on the quality of the recovered image via the compared schemes. For this purpose, the test color face images are attacked by various attacks that are listed in Table 4. Then, the compared schemes are executed for recovering the original content of the test images. Next, the average PSNR value is calculated for each scheme in each attack and is listed in Table 4. From this table, we can notice that the proposed scheme achieves higher quality of the recovered color face images compared to the other schemes for all attack types. This superiority can be explained by the fact that the proposed method involves the use of pre-trained deep learning models in the post-processing phase. This phase is undertaken to achieve better quality and more accurate colorization of the recovered face images.

In the next comparison, the key features of the proposed scheme are compared to a competing ones, which are presented in [3,4,8,25]. The comparison results are listed in Table 5. The latter indicates that the proposed scheme is pixel-based for watermark and recovery data embedding, which explains its superior accuracy in detecting tampered zones, as shown in Table 5. The proposed scheme also has a large key space of about $2^{494}$, which makes it robust against brute-force attacks by using modern computers. By contrast, the competing schemes do not provide information about their key space, which can make them vulnerable to brute-force attacks. Note that the “-” in Table 5 indicates that the information is not available in the original research article. Table 5 also indicates that our scheme uses pre-trained deep learning models to improve the quality of the recovered color face image, while the compared schemes do not, which explain the superiority of our scheme in comparison the compared ones in terms of the visual quality of the recovered color face images. Furthermore, the proposed scheme and the one presented by Al-Otum et al. [25] are both robust to salt-and-pepper noise. However, these schemes are not robust to Gaussian noise. This limitation needs further research to be overcome in future work.

Table 5 shows the average execution time in seconds for watermarking and recovery data embedding phase as well as for tamper detection and data recovery process. For this purpose, 2000 test images are arbitrarily selected from the datasets [40,46]. The selected images are then resized to 512 × 512 and attacked by irregular cropping attack with proportion up to 30%. Next, the average time of each phase is computed in seconds and then reported in Table 5. Based on the runtime comparison, our scheme outperforms the competing ones in watermark and recovery data insertion. This superiority is explained by the fact that AuCFSR incorporates watermark and recovery data in the spatial domain using low-complexity algorithms. However, our scheme is slower than the compared schemes at recollecting color face data. This drawback is explained by the fact that it runs two deep learning models: CodeFormer and DeOldify. This considerably increases the overall time taken to recover the altered data. This is an open problem for future research.

## 6. Conclusions

In this paper, we have presented a new two-dimensional chaotic system called 2D-MSCM. The rich chaotic behavior of this map is highlighted and its superiority over recent excellent 2D chaotic maps is also proven. Then, based on the suggested 2D-MSCM and deep learning models, a new scheme called AuCFSR for color face image tampering detection and self-recovery is introduced based on the proposed chaotic map and deep learning models. The MSCM 2D is used both to ensure the high level of security of the scheme provided and for the watermarking of fragile images. The obtained result demonstrated the good performance of the proposed scheme to obtain a high-quality color face image after watermark and recovery data integration. Moreover, AuCFSR has proven its high accuracy in detecting altered areas in the color face image and recovering altered areas through the use of pre-trained deep learning models. The comparative results provided a clear indication on the superiority of our scheme over recent ones in terms of tampering detection precision, security level and quality of the recovered color face images. In future work, the suggested scheme will be implemented in the transformation domain to compare its performance with the spatial ones. Moreover, other deep learning models will be invested and compared with those used in this work for selecting the best ones in the application of color image face tampering detection and self-recovery.

## Figures and Tables

**Table 1:** Related work concerning image tampering detection and self-recovery schemes with their characteristics.

Scheme’s Reference	Spatial/ Transform Domain	Block/Pixel-Based	Embedding Data Position (8 Bit Deep)	Image Category	Addressing the Tamper Coincidence Problem	Analysis of the Security Level?	Use of Deep Learning Techniques?
Aminuddin et al. [3]	Spatial	Block-based	2-LSB	Color	Yes	No	No
Aminuddin et al. [4]	Spatial	Block-based	2-LSB	Color	Yes	No	No
Molina-Garcia et al. [8]	Spatial	Block-based	2-LSB	Color	Yes	No	No
Tong et al. [18]	Spatial	Block-based	3-LSB	Color	No	No	No
Singh et al. [19]	DCT Transform	Block-based	3-LSB	Grayscale/Color	Yes	No	No
Cao et al. [20]	Spatial	Block-based	2-LSB	Grayscale	Yes	No	No
Tai et al. [21]	DWT Transform	Block-based	2-LSB	Grayscale	Yes	Yes	No
Fan et al. [22]	Spatial	Block-based	2-LSB	Grayscale	No	No	No
Bolourian Haghighi et al. [23]	LWT transform	Block-based	2-LSB	Grayscale/Color	No	No	No
Li et al. [24]	Spatial	Block-based	2-LSB	Grayscale	No	No	No
Al-Otum et al. [25]	Hybrid (spatial and DWT transform)	Block-based	2-LSB	Color	Yes	No	No
Su et al. [26]	Spatial	Block-based	2-LSB	Medical Grayscale	Yes	No	No
Rezaei et al. [27]	DCT Transform	Block-based	2-LSB	Grayscale	No	No	Yes
Proposed	Spatial	Pixel-based	2-LSB	Color Face image	Yes	Yes	Yes

**Table 2:** Average PSNR values of 5000 recovered color face images from the dataset [46] by using the proposed scheme.

Attacks	Proportion of Attacked Image Area				
	5%	10%	15%	20%	25%
Cropping	42.0127	39.3090	36.3278	32.1698	28.1234
Copy-move	43.1398	40.811	35.7809	33.0643	27.1678
Face swapping	42.5678	38.9865	35.1245	31.2236	28.5567
Object addition	43.4567	38.1289	35.4887	32.0097	26.1309

**Table 3:** Comparison in terms of average accuracy values of the compared schemes in detecting tampered areas by copy-move attack.

Scheme		Proportions of Copied-Moved Areas				
		10%	20%	40%	60%	80%
Schemes	Proposed	0.9988	0.9983	0.9978	0.9977	0.9976
	Aminuddin et al. [3]	0.9852	0.9806	0.9736	0.9719	0.9702
	Aminuddin et al. [4]	0.9852	0.9806	0.9736	0.9719	0.9702
	Molina-Garcia et al. [8]	0.9516	0.9632	0.9502	0.9703	0.9695
	Al-Otum et al. [25]	0.9420	0.9302	0.9455	0.9560	0.9400

**Table 4:** Comparison in terms of average PSNR values corresponding to the self-recovered images by the compared schemes.

Attacks	Schemes				
	Proposed	Aminuddin et al. [3]	Aminuddin et al. [4]	Molina-Garcia et al. [8]	Al-Otum et al. [25]
Cropping (rate of 25%)	28.3616	24.9063	25.1633	23.1613	21.3685
Cropping (rate of 50%)	23.0625	21.1696	21.635	20.1632	18.7452
Copy-move (rate of 25%)	27.3611	24.1696	25.1698	23.6354	22.1696
Copy-move (rate of 50%)	23.5063	21.1596	21.0056	20.1785	19.6321
Face swapping (rate 25%)	26.8852	24.0258	24.9820	22.9621	20.1633
Face swapping (rate 50%)	22.9630	20.9523	20.9816	20.1632	18.6592
Object addition (20% rate)	32.1622	29.1652	30.1487	27.1632	26.1233
Object addition (40% rate)	29.6305	27.0029	27.1598	25.1436	24.1678

**Table 5:** Comparison between the main features of the proposed scheme and similar ones.

Scheme Features	Schemes				
	Proposed	Aminuddin et al. [3]	Aminuddin et al. [4]	Molina-Garcia et al. [8]	Al-Otum et al. [25]
**Watermarking method** **(block-based/Pixel-based)**	Pixel-based	Block-based	Block-based	Block-based	Block-based
**Key space**	$2^{494}$	-	-	-	-
**Use of deep learning models?**	Yes	No	No	No	No
**Data embedding domain**	Spatial	Spatial	Spatial	Spatial	Hybrid
**Salt-and-pepper noise robustness?**	Yes	-	-	-	Yes
**Gaussian noise robustness?**	No	-	-	-	No
**Average runtime for watermarking and recovery data embedding**	1.0512	3.2346	2.1754	4.6580	5.9561
**Average runtime for tamper detection and data recovery**	15.7416	9.1644	10.3498	10.1230	12.1678

## Data Availability

All data will be available upon reasonable request.

## References

[1] Ray A., Roy S. (2020). Recent Trends in Image Watermarking Techniques for Copyright Protection: A Survey. Int. J. Multimed. Inf. Retr..

[2] Tolosana R., Rathgeb C., Vera-Rodriguez R., Busch C., Verdoliva L., Lyu S., Nguyen H.H., Yamagishi J., Echizen I., Rot P., Rathgeb C., Tolosana R., Vera-Rodriguez R., Busch C. (2022). Future Trends in Digital Face Manipulation and Detection. Handbook of Digital Face Manipulation and Detection: From DeepFakes to Morphing Attacks.

[3] Aminuddin A., Ernawan F. (2022). AuSR1: Authentication and Self-Recovery Using a New Image Inpainting Technique with LSB Shifting in Fragile Image Watermarking. J. King Saud Univ.-Comput. Inf. Sci..

[4] Aminuddin A., Ernawan F. (2022). AuSR2: Image Watermarking Technique for Authentication and Self-Recovery with Image Texture Preservation. Comput. Electr. Eng..

[5] Liu T., Yuan X. (2021). A Dual-Tamper-Detection Method for Digital Image Authentication and Content Self-Recovery. Multimed. Tools Appl..

[6] Molina J., Ponomaryov V., Reyes R., Sadovnychiy S., Cruz C. (2020). Watermarking Framework for Authentication and Self-Recovery of Tampered Colour Images. IEEE Lat. Am. Trans..

[7] Faheem Z.B., Ali M., Raza M.A., Arslan F., Ali J., Masud M., Shorfuzzaman M. (2022). Image Watermarking Scheme Using LSB and Image Gradient. Appl. Sci..

[8] Molina-Garcia J., Garcia-Salgado B.P., Ponomaryov V., Reyes-Reyes R., Sadovnychiy S., Cruz-Ramos C. (2020). An Effective Fragile Watermarking Scheme for Color Image Tampering Detection and Self-Recovery. Signal Process. Image Commun..

[9] Singh D., Shivani S., Agarwal S., Agrawal A., Tripathi R.C., Do E.Y.-L., Tiwari M.D. (2013). Self-Embedding Pixel Wise Fragile Watermarking Scheme for Image Authentication. Proceedings of the Intelligent Interactive Technologies and Multimedia.

[10] Kamili A., Hurrah N.N., Parah S.A., Bhat G.M., Muhammad K. (2021). DWFCAT: Dual Watermarking Framework for Industrial Image Authentication and Tamper Localization. IEEE Trans. Ind. Inform..

[11] Lee T.-Y., Lin S.D. (2008). Dual Watermark for Image Tamper Detection and Recovery. Pattern Recognit..

[12] Wang N., Zhang Y., Zhang L. (2021). Dynamic Selection Network for Image Inpainting. IEEE Trans. Image Process..

[13] Elharrouss O., Almaadeed N., Al-Maadeed S., Akbari Y. (2020). Image Inpainting: A Review. Neural Process Lett..

[14] Qin Z., Zeng Q., Zong Y., Xu F. (2021). Image Inpainting Based on Deep Learning: A Review. Displays.

[15] Wei Z., Min W., Wang Q., Liu Q., Zhao H. (2022). ECNFP: Edge-Constrained Network Using a Feature Pyramid for Image Inpainting. Expert. Syst. Appl..

[16] Zhou S., Chan K., Li C., Loy C.C. (2022). Towards Robust Blind Face Restoration with Codebook Lookup Transformer. Adv. Neural Inf. Process. Syst..

[17] Jantic J. (2019). Deoldify. GitHub: Github.com/jantic/DeOldify. https://github.com/jantic/DeOldify.

[18] Tong X., Liu Y., Zhang M., Chen Y. (2013). A Novel Chaos-Based Fragile Watermarking for Image Tampering Detection and Self-Recovery. Signal Process. Image Commun..

[19] Singh D., Singh S.K. (2016). Effective Self-Embedding Watermarking Scheme for Image Tampered Detection and Localization with Recovery Capability. J. Vis. Commun. Image Represent..

[20] Cao F., An B., Wang J., Ye D., Wang H. (2017). Hierarchical Recovery for Tampered Images Based on Watermark Self-Embedding. Displays.

[21] Tai W.-L., Liao Z.-J. (2018). Image Self-Recovery with Watermark Self-Embedding. Signal Process. Image Commun..

[22] Fan M., Wang H. (2018). An Enhanced Fragile Watermarking Scheme to Digital Image Protection and Self-Recovery. Signal Process. Image Commun..

[23] Bolourian Haghighi B., Taherinia A.H., Harati A. (2018). TRLH: Fragile and Blind Dual Watermarking for Image Tamper Detection and Self-Recovery Based on Lifting Wavelet Transform and Halftoning Technique. J. Vis. Commun. Image Represent..

[24] Li Y., Song W., Zhao X., Wang J., Zhao L. (2019). A Novel Image Tamper Detection and Self-Recovery Algorithm Based on Watermarking and Chaotic System. Mathematics.

[25] Al-Otum H.M., Ellubani A.A.A. (2022). Secure and Effective Color Image Tampering Detection and Self Restoration Using a Dual Watermarking Approach. Optik.

[26] Su G.-D., Chang C.-C., Lin C.-C. (2020). Effective Self-Recovery and Tampering Localization Fragile Watermarking for Medical Images. IEEE Access.

[27] Rezaei M., Taheri H. (2022). Digital Image Self-Recovery Using CNN Networks. Optik.

[28] Daoui A., Karmouni H., Sayyouri M., Qjidaa H. (2022). Efficient Methods for Signal Processing Using Charlier Moments and Artificial Bee Colony Algorithm. Circuits Syst. Signal Process..

[29] Jiang F., Tao W., Liu S., Ren J., Guo X., Zhao D. (2017). An End-to-End Compression Framework Based on Convolutional Neural Networks. IEEE Trans. Circuits Syst. Video Technol..

[30] Xu Q., Sun K., Cao C., Zhu C. (2019). A Fast Image Encryption Algorithm Based on Compressive Sensing and Hyperchaotic Map. Opt. Lasers Eng..

[31] Gao X. (2021). Image Encryption Algorithm Based on 2D Hyperchaotic Map. Opt. Laser Technol..

[32] Chen L., Tang S., Li Q., Zhong S. (2018). A New 4D Hyperchaotic System with High Complexity. Math. Comput. Simul..

[33] Zheng L., Zhang Y., Thing V.L.L. (2019). A Survey on Image Tampering and Its Detection in Real-World Photos. J. Vis. Commun. Image Represent..

[34] Christlein V., Riess C., Jordan J., Riess C., Angelopoulou E. (2012). An Evaluation of Popular Copy-Move Forgery Detection Approaches. IEEE Trans. Inf. Forensics Secur..

[35] Schetinger V., Oliveira M.M., da Silva R., Carvalho T.J. (2017). Humans Are Easily Fooled by Digital Images. Comput. Graph..

[36] Yee K., Tantipongpipat U., Mishra S. (2021). Image Cropping on Twitter: Fairness Metrics, Their Limitations, and the Importance of Representation, Design, and Agency. Proc. ACM Hum.-Comput. Interact..

[37] Korshunova I., Shi W., Dambre J., Theis L. Fast Face-Swap Using Convolutional Neural Networks. Proceedings of the 2017 IEEE International Conference on Computer Vision.

[38] Nguyen T.T., Nguyen Q.V.H., Nguyen D.T., Nguyen D.T., Huynh-The T., Nahavandi S., Nguyen T.T., Pham Q.-V., Nguyen C.M. (2022). Deep Learning for Deepfakes Creation and Detection: A Survey. Comput. Vis. Image Underst..

[39] Zhang X., Wang S. (2007). Statistical Fragile Watermarking Capable of Locating Individual Tampered Pixels. IEEE Signal Process. Lett..

[40] 70,000 Real Faces 2. https://www.kaggle.com/datasets/tunguz/70000-real-faces-2.

[41] Ekstrom M.P. (2012). Digital Image Processing Techniques.

[42] Zhou S. Sczhou/CodeFormer 2023. https://github.com/sczhou/CodeFormer.

[43] Kim C., Yang C.-N. (2021). Self-Embedding Fragile Watermarking Scheme to Detect Image Tampering Using AMBTC and OPAP Approaches. Appl. Sci..

[44] Siddiqui G.F., Iqbal M.Z., Saleem K., Saeed Z., Ahmed A., Hameed I.A., Khan M.F. (2020). A Dynamic Three-Bit Image Steganography Algorithm for Medical and e-Healthcare Systems. IEEE Access.

[45] FaceSwapper|Swap Photo Video Face Online Free. https://faceswapper.ai/.

[46] Flickr-Faces-HQ Dataset (FFHQ). https://www.kaggle.com/datasets/arnaud58/flickrfaceshq-dataset-ffhq.

[47] Boulogeorgos A.-A.A., Alexiou A., Merkle T., Schubert C., Elschner R., Katsiotis A., Stavrianos P., Kritharidis D., Chartsias P.-K., Kokkoniemi J. (2018). Terahertz Technologies to Deliver Optical Network Quality of Experience in Wireless Systems Beyond 5G. IEEE Commun. Mag..

[48] Daoui A., Mao H., Yamni M., Li Q., Alfarraj O., Abd El-Latif A.A. (2023). Novel Integer Shmaliy Transform and New Multiparametric Piecewise Linear Chaotic Map for Joint Lossless Compression and Encryption of Medical Images in IoMTs. Mathematics.

[49] Alvarez G., Li S. (2006). Some Basic Cryptographic Requirements for Chaos-Based Cryptosystems. Int. J. Bifurc. Chaos.

